# From virtually extinct to superabundant in 35 years: establishment, population growth and shifts in management focus of the Swedish wild boar (*Sus scrofa*) population

**DOI:** 10.1186/s40850-024-00202-2

**Published:** 2024-07-01

**Authors:** Göran Bergqvist, Jonas Kindberg, Bodil Elmhagen

**Affiliations:** 1 Swedish Association for Hunting and Wildlife Management, Öster Malma, Nyköping, SE-611 91 Sweden; 2 Southern Swedish Forest Research Centre, Faculty of Forest Sciences, Swedish University of Agricultural Sciences, PO Box 49, Alnarp, SE-230 53 Sweden; 3 Department of Wildlife, Fish and Environmental Studies, Faculty of Forest Sciences, Swedish University of Agricultural Sciences, Umeå, SE-901 83 Sweden; 4 Nowegian Institute for Nature Research, PO Box 5685, Torgarden, Trondheim, NO-7485 Norway; 5 Department of Zoology, Stockholm University, Stockholm, SE-106 91 Sweden

**Keywords:** Act, Commission, Exponential, Legislation, Logistic, Ordinance

## Abstract

**Background:**

The wild boar (*Sus scrofa*) was extinct in Sweden when a few animals established in the 1970s. Over the past 35 years, the species has made a substantial comeback. In this paper, we analyse wild boar population growth using three indices of population size. We also map the legislative decisions and research prompted by the expanding population. We discuss to what extent, in the eyes of the state, the view of wild boar and the management focus has shifted over time, from a perceived pest (eradication) to scarce (conservation), overabundant (reduction/control) or somewhere in between (sustainable management).

**Results:**

Wild boar harvest started in the early 1990s with a few hundred animals annually and peaked at 161,000 in 2020/2021. The distribution now comprises most of southern Sweden. Analyses of harvest and traffic accidents involving wild boar showed that the population grew exponentially until 2010/2011, after which the increase levelled off. Thus, logistic growth models showed the best fit for the full study period. We recorded 38 legislative decisions or commissions to government agencies regarding wild boar. The first decision in 1981 was to eradicate the free-ranging population. In 1987 however, the parliament decided that wild boar is native to Sweden and should be allowed in restricted extent. Later decisions mainly concerned hunting regulations and hunting methods as direct means to increase harvest and regulate the population. Another topic, increasing in importance over time, was to facilitate the use of wild boar meat to indirectly stimulate harvest. A local outbreak of African swine fever in 2023 necessitated a stamping out strategy in the affected area. We found 44 scientific papers regarding the present free-ranging population. Topics include movements and feeding patterns, hunting, reproduction, and population development.

**Conclusions:**

The state historically regarded wild boar as a pest to be eradicated. This changed with the decision that wild boar should be allowed in restricted extent, suggesting a conservation approach. In response to population growth, the focus shifted to means facilitating sustainable management and, lately, reducing growth. The story of wild boar in Sweden illustrates attempts to mitigate conflicts and balance interests in wildlife management.

**Supplementary Information:**

The online version contains supplementary material available at 10.1186/s40850-024-00202-2.

## Background

Biological diversity is presently decreasing at the global scale due to drivers such as anthropogenic land use, exploitation through hunting/fishing/trapping and competition from invasive species [1, 2]. For instance, the global biomass of wild mammals has decreased by 82% since pre-historic time [3]. However, political goals are currently in operation with the aim of reversing this trend of decreasing biological diversity [4]. Underlying reasons for this goal may be ethical considerations, awareness of biological diversity as a resource (ecosystem services), or an increasing awareness of the function of different species in the ecosystem. As a result, conservation measures may be taken where species are reintroduced or allowed to recolonise habitats. For some species, however, the original decline or local extinction was intentional, an outcome of conscious decisions aimed at reducing conflicts of interests, for instance to mitigate damage to agricultural crops [5] or depredation on livestock. Well-known examples of such intentional extirpation regarding large carnivores are reviewed by Ripple et al. [6]. Following the return of such species, the original human-wildlife interaction may resurface, and mitigation of the conflict may again become a challenge to wildlife management. In this article, we exemplify this process with the return of the wild boar (*Sus scrofa*) in Sweden. We analyse how the Swedish wild boar population has expanded in time and space, and review and discuss how the consequent trade-offs in the human-wildlife interaction are manifested in political decisions and research.

In contrast to species that have declined due to reduced availability or quality of suitable habitats, the habitats of intentionally extirpated species may still be highly suitable. When such species are reintroduced or no longer supressed at a low level, the conditions may favour a rapid population growth and recovery. In theory this means that the population may grow exponentially during an initial phase, and then revert to a lower growth rate as the ecological carrying capacity is approached [7], or to the species becoming again actively controlled due to resurgent conflict with human interests. This also applies when a species is introduced to a completely new environment and becomes invasive, as well as for native species that find a new niche in a new habitat [8]. For instance, several goose species that earlier showed declining populations have become superabundant, partly due to a shift in foraging from natural habitats to an increased use of agricultural landscapes [9]. At some point, a population may even be considered overabundant and a pest. A species can be regarded as overabundant when individuals of that species, (a) affect human life or well-being, (b) affects the fitness of the overabundant species, (c) reduce the population density of species with an economic or aesthetic value, or (d) cause dysfunction in the ecosystem [10]. Thus, as the nature of human-wildlife interactions may be density-dependent, the desired focus of management can quickly change as a population recovers, from conservation at low densities, to sustainable management, to reduction of the species in question at higher population densities [11].

The wild boar is sometimes regarded as a pest. After being absent or only occurring at low population densities, it has increased greatly, both in numbers and distribution in many parts of Europe during the latest decades [12], and its conservation status is classified as least concern by the IUCN Red List [13]. Wild boar has also been introduced to many areas outside of its native range and is now one of the most widely distributed mammal species in the world, present on all continents except Antarctica [14]. There are several factors contributing to this expansion. The wild boar is an opportunistic omnivore that consumes a wide variety of food items [15], and it efficiently exploits increased or pulsed resources [16, 17]. Given sufficient food resources, females start breeding early in life [18], and produce large litters [19–21], resulting in the highest reproductive potential of all European ungulates [22]. Wild boar also show tolerance to harsh climatic conditions and inhabit areas as far north as 64 ^o^N in Eurasia [23].

Wildlife, including wild boar, provides a range of ecosystem services that are beneficial to humans, but also disservices that are disadvantageous [11]. The wild boar is regarded as an ecosystem engineer and can have strong effects on ecosystem functioning through its ability to create, modify and/or destroy habitats for other species [24]. The outcome of a certain activity, such as wild boar rooting, may depend on the extent of the activity which, in turn, may depend on the number of animals. At low population densities, the outcome of an activity such as rooting may be minor [25] or positive, such as increased species richness [26]. During such conditions, management decisions and/or topics of research may aim at preserving or increasing the species [11]. For instance, re-introduction of wild boar has been discussed as a tool to restore the Caledonian pine forest in the Scottish Highlands by reinvigorating the disturbance regime [27]. However, agricultural crops constitute an important component of wild boar diet in its native range [15, 28], resulting in damage to farmlands [29, 30]. Furthermore, the cost of vehicle accidents involving wild boar can be high, for example it is estimated to EUR 9.7–12.3 million annually in Sweden [31]. Additionally, both wild boar and domestic pigs can be infected by African swine fewer (ASF), a viral disease that has spread through large parts of Europe and Asia since 2007 and that constitutes a major threat to pig production and the pork industry. Outbreaks in the wild boar population are difficult to combat and ASF may become endemic in wild boar populations, contributing to the spread of this disease [32, 33]. Thus, as wild boar population density increases, there are several reasons why management decisions may shift focus from conservation towards population limitation [11]. Topics of management and/or research interest may then include aspects of different hunting methods [34–37], monitoring methods [38], or the extent of damage, a topic where numerous studies have been carried out [14, 39]. Especially in its introduced range, where wild boar is classified as one of the 100 worst invasive species [40], management decisions may strive for eradication and include methods that would normally not be allowed for native species, such as shooting from the air or poisoning [41].

In Sweden, wild boar is a native species, but it was eradicated due to historic human-wildlife conflicts [42, 43]. Geological findings show that the species was present in parts of southern-to-central Sweden already during the Mesolitic and Neolitic time-periods. It was subject to heavy hunting and also assimilated into domestic pig populations [44]. It is not known exactly when wild boar became extinct because it is difficult to distinguish between wild boar and feral pigs, including individuals with hybrid origin, in archaeological findings [45]. However, from around 1000 A.D. there is no certain evidence for anything but local and/or temporary populations [42]. For example, wild boar was present and hunted on the large (1300 km^2^) island Öland in the late 17th century. The Öland population then disappeared, but wild boar was reintroduced on the island in the 18th century by the royal court for hunting purposes. However, it is known that the reintroduced population was actively eradicated after some time due to agricultural damages [46]. Small and local populations of wild boar have since established in Sweden from time to time, but these populations were also eradicated after local complaints, for instance regarding hybridisation with domestic pigs and damage to agricultural crops [47, 48]. The present free-ranging wild boar population in Sweden was not intentionally re-introduced, i.e., there was no government decision to re-introduce the species, but the population stem from escapees and possibly deliberate releases from enclosures, especially during the 1970s and 1980s [49].

In this paper, we assess how the development of the wild boar population in Sweden relates to the associated development in wild boar management and knowledge needs. Specifically, we (1) analyse the temporal and spatial population development and test whether the population growth has been exponential and/or has started to level off at the national level. We (2) review the political decisions regarding wild boar management that have been taken over time, and (3) the development of knowledge needs as illustrated by research topics of published scientific papers over time. We discuss whether wild boar and the associated management strategy, in the eyes of the state, falls into the category of species that are considered scarce (management for conservation) or overabundant (strong reduction and/or control), or somewhere in between (sustainable management) and whether this has shifted over time.

## Methods

### Study area

The distribution of the free-ranging wild boar population under study comprises all counties in southern and central Sweden, except the islands of Gotland and Öland in the Baltic Sea. The area includes hemiboreal, nemoral and southern boreal vegetation zones [50], spanning approximately latitudes 55.3 ^o^N to 62.2 ^o^N and longitudes 11.1 ^o^E to 19.1 ^o^E. Agricultural land, pasture and forests cover approximately 23,000, 4,000 and 129,000 km^2^, respectively [51]. Ley (e.g., clover *Trifolium* sp. and timothy *Phleum pratense*), wheat (*Triticum aestivum*), barley (*Hordeum vulgare*) and oats (*Avena sativa*) are common agricultural crops [52]. Forests are dominated by Scots pine (*Pinus sylvestris*), Norway spruce (*Picea abies*) and birch (*Betula pendula* and *B. pubescens*). Oak (*Quercus robur*) occurs throughout the area, and beech (*Fagus sylvatica*) occurs in the southernmost part, but only in small amounts [53]. Supplementary feeding of game species is legal in Sweden and is common all year around.

### Background facts about hunting in Sweden

In Sweden, the right to hunt belongs exclusively to the landowner. This has been the case since year 1789 when King Gustav III granted all landowners this right [54]. At present, approximately 50% of all land in Sweden is privately owned, 15% is owned by the state, and the remaining 35% by private companies or the church [55]. Landowners have free disposition of their hunting rights and may use it personally, decide not to use it, or lease it to someone else.

The general acceptance for hunting among the Swedish population is high, as around 80% of the non-hunters accept or advocate hunting [56]. A potential reason is that most of the population was rural until urbanisation accelerated in the early 20th century. A large proportion of the population has therefore experienced hunting, either themselves or by knowing a hunter, and they have access to game meat which is associated with a positive view of hunting [56].

To become a hunter in Sweden, one must pass a hunting exam that consists of theoretical and practical tests, including safe weapons handling and shooting tests. The exam is valid for life. After a person has passed the exam, they can apply to the police for a weapons license, and a valid license is required to hunt with a weapon. Finally, to actively hunt in a particular year, the hunter must pay a yearly game management fee, and then become registered as a hunter. Approximately 2.7% of the 10.5 million inhabitants in Sweden are registered as hunters [57], equivalent to 0.5 hunters per each km^2^ land area.

The organisation for hunting is largely built around the moose (*Alces alces*), the largest ungulate species in Sweden and traditionally the most important game species. Initially, the aim of organising hunting was to achieve a sustainable management of this species [58], which was over-harvested and close to extinction in the 19th century. As a result of the moose hunting organisation, it is common that several landowners and/or hunters lease hunting rights for an area where they form a hunting team and hunt together. Hunting teams are generally geographically stable, as many hunters are landowners or lease hunting rights close to their place of residence or in an area where they have an attachment to, e.g., through relatives. It is therefore common that the same families have hunted in a specific area for many generations although, parallel to this, urbanisation and modernisation where a developed infrastructure facilitates travelling has also increased the number of hunters who lease hunting in places that they lack a previous attachment to.

Depending on the species to be hunted and time of the year, a variety of hunting methods are allowed, such as still hunting, drive hunts with or without dogs, hunting with dogs that bark at bay, stalking and trapping. According to Swedish tradition, dogs are frequently used in hunting, but only one or a few dogs on each hunting occasion. This is different from many other countries where larger packs of dogs are commonly used [59].

Sweden joined the European Union (EU) in 1995. This has resulted in the Swedish hunting legislation being adapted to European legislation, in particular the Birds Directive (79/409/EEC, 2009/147/EC) and the Habitats Directive (92/43/EEC).

### The Swedish Association for Hunting and Wildlife Management (SAHWM)

SAHWM was founded in 1830 and is the largest of the Swedish hunting associations. It is a non-government organisation (NGO), but since 1938 the Swedish government has given SAHWM an annual commission to organise and direct parts of the game management and hunting in Sweden, and in recent years the wild boar has been a prioritised species. The commission also includes an assignment to estimate the annual harvest of game species without mandatory reporting, such as wild boar. Hence, in Sweden, much of the information and training regarding hunting and wildlife management comes from a hunters´ organisation, rather than from a government body, and hunters also report their harvest to the same organisation. To the best of our knowledge, this model is unique for Sweden [60].

### Crop damages

In Sweden, there is no system for economic compensation of damages caused by game with an open hunting season. Since landowners owns the hunting right, they are expected to adjust harvest levels in order to avoid excessive damages.

Data on damages on agricultural crops are collected by different NGOs representing farmers, but also by The Swedish Board of Agriculture (SBA). They reported that the loss of crops to game amounted to 3.1% of the expected harvest in 2020, and that wild boar was the main culprit [61].

### Wild boar population data

In Sweden, there is no national monitoring program for wild boar. To assess wild boar population growth spatially and temporally we used three independently collected time series: (1) estimated harvest, (2) the number of *Trichinella* tests carried out on harvested wild boars intended for consumption, and (3) the number of traffic accidents involving wild boar. At the national level, the number of *Trichinella* tests was compared to the estimated harvest as an indication of the quality of the harvest estimates. Data on estimated harvest, which has the highest spatial distribution, was used to assess the spatial expansion of the wild boar distribution. To assess population growth over time, including testing whether wild boar population growth was exponential or logistic, we used data on estimated harvest and traffic accidents involving wild boar as proxies for population size.

#### Estimated harvest

In Sweden, harvest reporting is voluntary for most game species, including wild boar. Hunting teams report their harvest of game species, along with the size and location of their hunting ground, and the total harvest at larger spatial scales is estimated based on these reports. Harvest was previously reported for the period April 1 to March 31, but in 2016/2017 the period was changed to the official hunting year, July 1 to June 30 of the following year, although for most native species in Sweden, the open season only comprise part of the hunting year.

Wild boar was included in the harvest reporting from the hunting year 1990/1991. Up until 1995/1996, the regional staff at SAHWM were responsible for estimating harvest in their respective counties (*N* = 21), which meant that the methods applied may have differed slightly among counties. However, a basic approach would have been to extrapolate the voluntarily reported number of harvested wild boar, based on the proportion of the reported area in relation to the total area, hence calculating a point estimate for the county. In this paper we use data calculated as point estimates for the period 1990/1991 to 1996/1997.

The method to collect data and estimate harvest changed in 1995/1996 so that harvest was estimated at the smaller spatial scale of Hunting Management Precinct (HMP: the number has decreased slightly over time but *N* ≈ 325). The area for which hunting teams voluntarily have provided harvest reports varies slightly among years but is on average 29.9 ± 3.8 (mean ± SD) % of the total area for hunting in Sweden during the hunting years 1995/1996 to 2021/2022. In cases where the harvest report appears potentially incorrect, for example due to an unusually high/low reported harvest or the reported harvest of a species that would be rare or unlikely to be present in the area, the reporting person is contacted in order to straighten out any unclarities [62].

In this paper, all harvest estimates from 1997/1998 an onwards have been calculated with a method developed in recent years, building on Bayesian inference, and including the application of an autoregressive component [63, 64]. The reason why the new method was not applied on the harvest data already for 1995/1996 and 1996/1997 was that the development of the system resulted in insufficient data at the HMP level for those years. The resulting harvest estimates are Bayesian estimates, including a 95% credibility interval. The Bayesian estimates are based on a total of 148,038 individual harvest reports, corresponding to an average of 5,922 ± 1,149 (mean ± SD) reports per year. Harvest estimates are then available at three geographical units (spatial scales): HMPs, counties and national level. All population density estimates used in this paper was provided by SAHWM.

To use harvest as a proxy for population development is a common procedure, as harvest bags are among the most frequent data reported by hunters [65], and for many species it is the only data available. A key question is whether harvest bags correctly monitor population development, and reported results are divergent. For instance, harvest data was reported to accurately reflect changes in red grouse (*Lagopus lagopus scoticus*) population density in Great Britain [66]. On the other hand, patterns of population dynamics of three grouse species in Finland differed when based on harvest data as compared to census data, although data were positively and linearly related [67]. One important prerequisite for harvest data to accurately reflect population development is that there are no drastic or sudden changes in, e.g., hunting legislation or length of the open hunting season [68]. As wild boar in Sweden traditionally has the longest open hunting season of all ungulates, with few and small changes over time (see Results), we anticipate no such effects on data. Analysis of population development can be improved if additional and independent data series are available [69]. This is the case for wild boar in Sweden, as also the number of traffic accidents are available since 2003. The developmental patterns of these time-series are very similar and highly correlated (see Results). It is therefore reasonable to conclude that they accurately reflect the development of the wild boar population at the national level. However, indirect measures do not readily provide estimates of population size, only development. Management of the Swedish wild boar population could be further refined if also quantitative population estimates were available.

#### Establishment

From 2008/2009 and onwards, hunting teams have the opportunity to also report the degree of establishment of wild boar when they report their total harvest. Establishment was subjectively classified as one of three categories: no establishment, sporadic occurrence or established. In the counties under study, a total of 49,578 establishment reports were provided for the hunting years 2008/2009 to 2020/2021, corresponding to an average of 3,814 ± 537 reports per year.

For each hunting year and county, the proportion of the reported area for each degree of establishment was calculated. This proportion was then multiplied with the total huntable area of the county in question [70], and the annual change in the area with established wild boar populations was calculated.

#### Trichinella tests

*Trichinella* parasites can be transferred from animals to humans through undercooked meat and the resulting disease, trichinosis, may be lethal for humans. *Trichinella* occur sporadically in wild boar in Sweden, 3–9 cases per year in 2017–2021 [71]. For this reason, hunters in Sweden are only allowed to consume wild boar meat within their own household or sell it to designated facilities. It is voluntary to test wild boar meat for *Trichinella* when the meat is intended for household consumption, but it is believed that almost all hunters do this. When the wild boar is sold to a designated game meat handling facility, it is the responsibility of the facility to ensure that testing is performed. Starting in 2004, The Swedish Veterinary Agency (SVA) collects annual data on *Trichinella* tests from all accredited laboratories in Sweden, for reporting to the European Food Safety Agency (EFSA). However, data from one laboratory was missing in 2012–2014 and it is possible that some data were missing also in earlier years (2004–2011), but data are complete from 2015 and onwards (Lundén A, SVA, pers comm). *Trichinella* tests are reported by calendar year, and the data are published in annual reports [71]. From 2004 to 2021, a total of 1,237,835 *Trichinella* tests were reported, corresponding to an average of 68,769 ± 46,245 per year.

#### Traffic accidents

In Sweden, it is mandatory to report traffic accidents involving several wild species, including wild boar, to the police. Starting in 2003, data on the number of reported traffic accidents each month is publicly available for a number of species [72]. In this paper, traffic accidents are summed per hunting year, to enable direct comparison to estimated harvest (see above). A total of 70,479 traffic accidents involving wild boar were reported for the hunting years 2003/2004 to 2021/2022, corresponding to an average of 3,709 ± 2,147 per hunting year.

### Statistical analyses

To test whether the wild boar population grew exponentially, or if the growth rate levelled off during certain time periods, models for standard exponential and logistic growth were fitted to time series data with nls (non-linear least squares) following Stevens [73]. For comparison, model fitting was done for the two independent time series estimated harvest and traffic accidents. To test if the best fitting model changed over time, for example if there was a switch from exponential to logistic population growth at some point in time, model fitting was done for each time series as a whole, as well as for each time series gradually shortened by one year time steps.

The time-explicit equations for exponential and logistic growth fitted to data were.

Eq. 1. Exponential growth ${N_t} = {N_0}{e^{rt}}$

Eq. 2. Logistic growth ${N_t} = {N_0}{e^{rt}}/{\text{ }}(1 + \alpha {N_0}\left( {{e^{rt}}-{\text{ }}1} \right))$

Where *N* is population size, *t* is the time unit (hunting year), *r* is population growth rate and α = 1/*K* where *K* is the carrying capacity.

For each model and time period, nls was used to search for the parameter values (N0, r, α) which provided the best fit to the data [73]. The best exponential and logistic growth models were then compared with Analysis of Variance (ANOVA), where a significant difference implied that the logistic growth model provided a better fit to the data. When there was no significant difference between the fit of the two models, exponential growth was assumed, as the exponential model is the parsimonious (simpler) model.

From an ecological standpoint, a logistic growth model cannot have an α-value below 1 because this would suggest a carrying capacity below 0 (as K = 1/ α). Hence, when the best fitting logistic model rendered by nls had a negative α value, the model fitting process was re-run as a conditional nls where α < 0 was not allowed. Then, nls rendered logistic models with α = 0, which implies that the model is identical to an exponential model (ANOVA, *p* = 1.0) and the exponential model was consequently assessed as the best, parsimonious, model.

Spearman rank correlations (r_s_) were used to test the strength of the correlation between estimated harvest, traffic accidents and *Trichinella* tests.

All tests were performed in R 3.6.3 [74].

### Reviews

Management of game species involves political decisions to steer management and population development in a desired direction. It also involves the build-up of knowledge needed in management. To assess how the development of the free-ranging wild boar population under study has been guided by political decisions and new knowledge, we reviewed legislative decisions and commissions to government agencies, as well as published scientific papers.

#### Legislative decisions and commissions

The nomenclature in this chapter follows the Multilingual dictionary from the Swedish Parliament [75].

Legislation in Sweden is documented in Acts and Ordinances. Acts are decided by the parliament and are superior documents, whereas Ordinances are decided by the government and usually contains detailed interpretations of the Act in question.

Starting in 1825, all Acts and Ordinances are given individual numbers in the Swedish Code of Statues (SFS). These are the official and authentic versions of Acts, Ordinances and government agency regulations. The same applies for all changes and additions, which receive individual SFS numbers. It is therefore possible to track all changes in Acts and Ordinances through their SFS numbers.

All SFS published between June 9, 1988, and March 29, 2018, can be found online [76] whereas all later SFS are published at [77]. Using these online sites, we checked all SFS relating to the present Hunting Act (SFS 1987:259) and Hunting Ordinance (SFS 1987:905) for changes or additions related to wild boar.

We then commissioned the library of the Swedish parliament to conduct two separate searches in their SFS database, one using the search term “vildsvin” (wild boar) and one using “jakt” (hunting), to find relevant SFS published prior to the online publications, and to validate our findings. These searches were conducted on February 16, 2023.

The parliament or government can also make decisions that do not result in a change in an Act or Ordinance, and therefore have no SFS number. Such decisions are referred to by its registration number. In addition, the government can also give commissions to government agencies in their annual appropriation directions.

On March 16, 2023, we sent e-mails to the registrar of the Environmental Protection Agency (Swedish EPA), Swedish Food Agency (SFA), SVA and SBA, respectively, asking them to specify all past and present government commissions regarding the present free-ranging wild boar population.

A government commission to a government agency typically results in a written response from the agency in question. Often the agency also appoints an external part, most often a university, to investigate different aspects of the commission. In our survey we only included the original commissions from the government, not the response from agencies, as our primary goal was to map the topics covered by government commissions.

The annual commission from the government to SAHWM was not specified in detail regarding content before 2005. Instead, broader areas of responsibilities were given such as “inform about hunting” or “estimate harvest of game species” and specific game species were usually not mentioned. From 2005 and onwards, the government instead divided the commission into ten categories, with specific tasks to be carried out within each category [78]. Examples of the categories are game monitoring, including harvest estimations, and to administer the nation-wide organisation to handle game that has been wounded by traffic. Often, a specific task would be ongoing for several years, and the task in question was then repeated in the commission each year. For instance, the task to provide information to hunters about the importance of testing for *Trichinella* was first mentioned in 2012 and included all years after that. In this study, we only report the first year, i.e., 2012 for information about *Trichinella*.

#### Published research

To determine the extent and topics of research carried out regarding wild boar in Sweden, we conducted a search on Web of Science on February 1, 2023, using the search terms “Wild boar” OR “Sus scrofa” AND “Sweden”. The search yielded a gross list of 271 published papers.

We evaluated the studies by reading the abstracts, then refined the list to only include studies that directly concerned the present free-ranging wild boar population in Sweden, or where wild boar in Sweden was one (or one of a few) focus species. For example, we excluded archaeological studies that concerned historic wild boar populations in Sweden, as well as studies where wild boar in many countries were sampled for a disease (i.e., the study aimed to assess the frequency or distribution of an infectious agent rather than a specific study of the Swedish wild boar population). This yielded a net list of 40 published studies. Based on personal knowledge, we added two studies that were not captured by the Web of Science search, increasing the number of included studies to 42.

We divided the studies into seven categories: health (including diseases), reproduction, movements (including natal dispersal and home ranges), feeding (including rooting and damages), hunting, traffic and population development, and noted the year for each publication. Furthermore, we classified each paper as focusing on damage/other adverse effects or describing ecology and/or management related issues.

## Results

### Spatial and temporal development

**Fig. 1:**
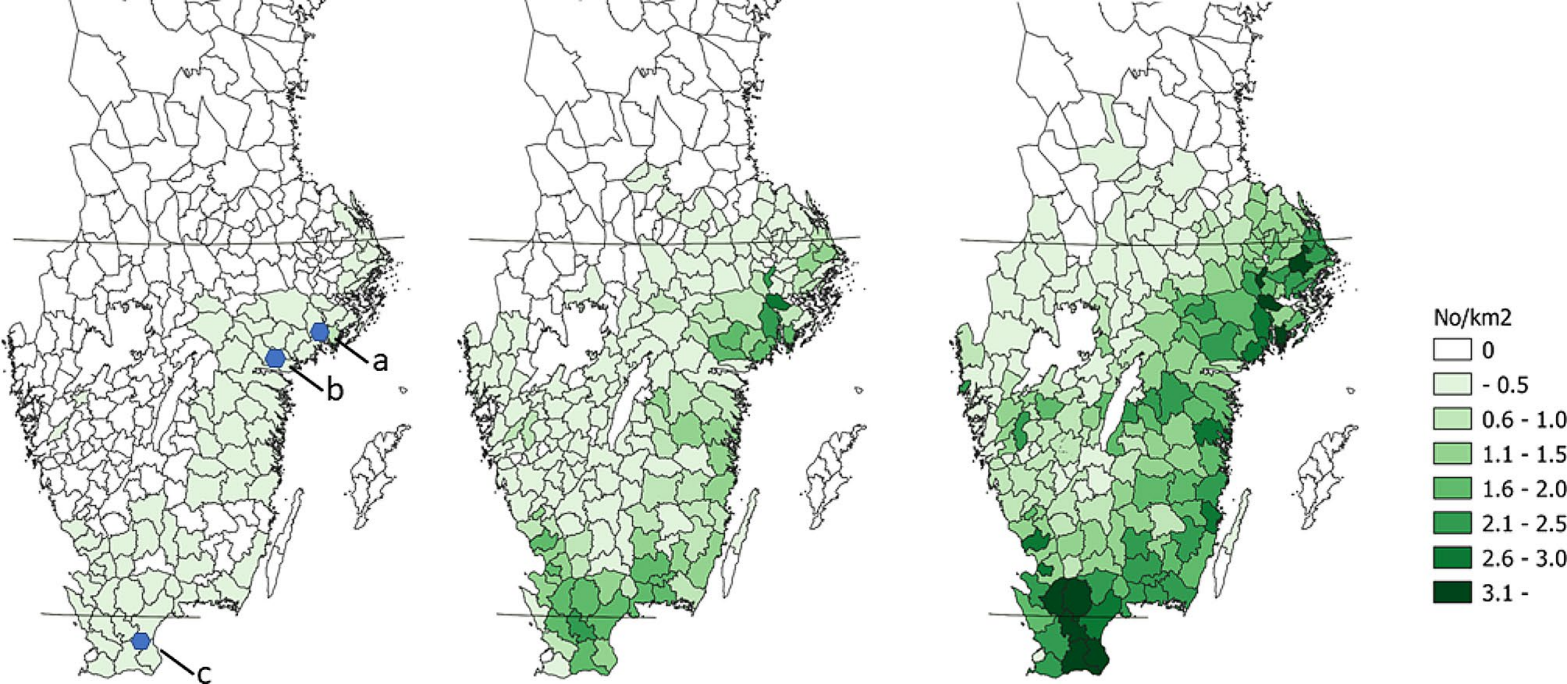
Estimated harvest of wild boar (no. per km^2^) per Hunting Management Precinct (HMP) for the hunting years 2000/2001 (left), 2010/2011 (middle) and 2020/2021 (right). Solid horizontal lines show latitudes 56 ^o^N and 60 ^o^N. Locations of the core areas, Tullgarn/Mörkö (a), Björkvik (b) and Linderödsåsen (c) are indicated in the leftmost map

The initial establishment of wild boar occurred in three core areas: Tullgarn/Mörkö in Stockholm/Södermanland counties, Björkvik in Södermanland county and Linderödsåsen in Skåne county, approximate locations indicated in Fig. 1. In February 1980, there was a sparse, free-ranging wild boar population consisting of 70–110 animals mainly in core area a, Fig. 1.

In 2000/2001, the mean estimated harvest was 0.03 animals per km^2^ (median 0, min 0, max 0.6) in the area under study. It increased to 0.4 per km^2^ (median 0.1, min 0, max 2.9) in 2010/2011 and further to 1.2 per km^2^ (median 1.0, min 0, max 5.6) in 2020/2021.

The reported harvest of wild boar was initially concentrated to areas in proximity to the core areas and expanded gradually towards the west and north (Fig. 1). For the period 2008/2009 to 2020/2021 the area with established populations increased by an average of 4,260 km^2^ annually, corresponding to 2.4% of the total huntable area in the counties under study.

**Fig. 2:**
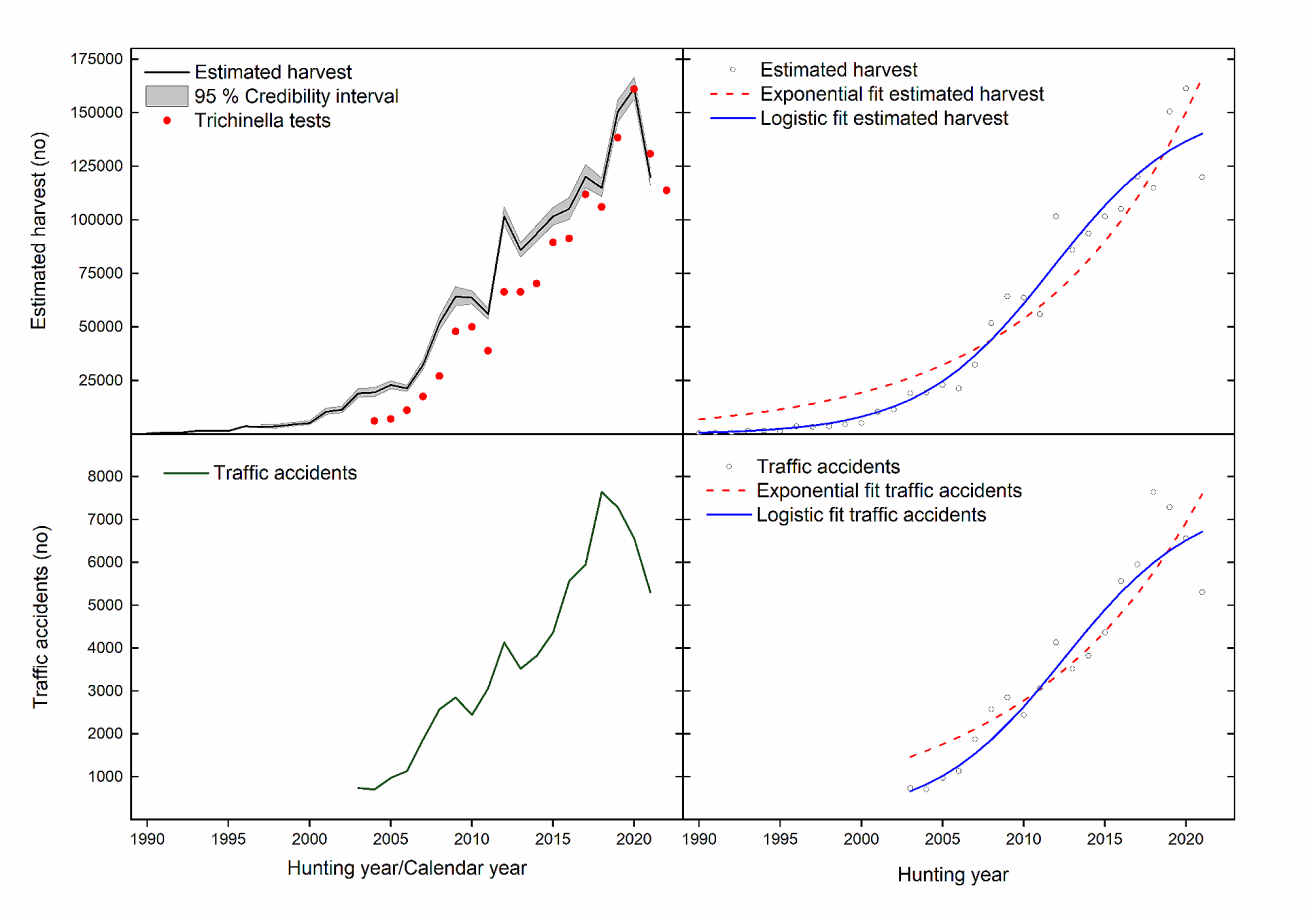
Upper left graph: estimated harvest (black line) with a 95% credibility interval (grey area from 1997/1998 and onwards) and *Trichinella* tests (red dots). Upper right graph: estimated harvest (circles) and the population growth models representing the overall best exponential fit (red dashed line), and the overall best logistic fit (blue solid line) to the harvest data. Lower left graph: traffic accidents involving wild boar (green line). Lower right graph: traffic accidents involving wild boar (circles) and the population growth models representing the overall best exponential fit (red dashed line), and the overall best logistic fit (blue solid line) to the traffic accidents data. Estimated harvest and traffic accidents are reported by hunting year and *Trichinella* tests by calendar year. Note differences in Y-axis scales between upper and lower graphs

Starting with a modest harvest of just 334 animals in 1990/1991, the annual estimated harvest has increased and peaked at 160,892 animals in 2020/2021. *Trichinella* tests show a similar pattern, increasing from 1,691 tests in 2004 to 161,072 tests in 2020, whereas traffic accidents involving wild boar increased from 734 in 2003/2004 and peaked at 7,641 accidents in 2018/2019. All datasets show a pronounced decrease during the last year of the study, in the case of traffic accidents during the last three years (Fig. 2). Estimated harvest, *Trichinella* tests and traffic accidents show similar development patterns and are all positively and significantly correlated (estimated harvest vs. traffic accidents, r_s_ = 0.96, *p* < 0.0001, estimated harvest vs. *Trichinella* tests, r_s_ = 0.99, *p* < 0.0001 and traffic accidents vs. *Trichinella* tests, r_s_ = 0.95, *p* < 0.0001). The overall ratio of estimated harvest to traffic accidents during 2003/2004 to 2020/2021 is 22:1.

Analyses of the fit of exponential and logistic growth models to the longest time series, estimated harvest from 1990/1991 to 2021/2022, suggest that wild boar harvest grew exponentially from 1990/1991 up until 2010/2011. When the time series was extended stepwise by additional years after 2010/2011 and up until 2021/2022, the logistic growth model generally provided a significantly better fit (for 10 of 11 added years: Additional file 1). Thus, for the study period as a whole (1990/1991 to 2021/2022) a logistic growth model provided the best fit to the harvest data (Fig. 2, Additional file 1).

Analyses of the fit of exponential and logistic growth models to the shorter time series for the traffic accidents involving wild boar, available from 2003/2004, suggest that the number of accidents grew exponentially from 2003/2004 up until 2010/2011. Extending the time series by additional years after 2010/2011, the logistic growth model generally provided a significantly better fit to the traffic accident data for some years (better fit for 3 out of 4 added years up until 2014/2015). After 2014/2015, there was a shift back to exponential growth that lasted until 2019/2020 (Additional file 1). For the study period as a whole (2003/2004 to 2021/2022), a logistic growth model provided the best fit for the traffic accidents data (Fig. 2, Additional file 1).

### Legislative decisions and commissions

#### Legislation from 1938 to 1980

The Hunting Act (SFS 1938:274) and Hunting Ordinance (SFS 1938:279) were active from 1938 until 1987, i.e., primarily before the present wild boar population was introduced. In the Hunting Act of 1938, wild boar is only mentioned in § 24, that gives the government, or an appointed government agency, the right to decide on measures to reduce or eradicate a local wild boar population. In the Hunting Ordinance of 1938, wild boar is only mentioned in § 9, granting the use of a fixed light, not primarily intended for hunting, in hunting of wild boar among other species.

During the first part of this time period, decrees were published annually, designating the time of the year when each game species was protected from hunting. Wild boar was not mentioned during this time (1939–1966, all SFS numbers given in Additional file 2). Only from 1967, the annual decrees started to designate the open hunting season for each game species. Wild boar is mentioned in § 1 as a species with open season all year around (SFS 1967:773, 1968:355, 1969:353, 1970:274, 1971:446, 1972:246, 1973:406, 1974:554). In 1975, the annual decrees were replaced by an annual Hunting Season Ordinance. As previously, wild boar is mentioned in § 1 as a species with open season all year around (SFS 1975:543, 1976:432, 1977:327, 1978:776). After 1978, SFS regarding hunting seasons were published at irregular intervals, when changes occurred.

#### Legislative decisions and commissions during 1981 to 2022

**Fig. 3:**
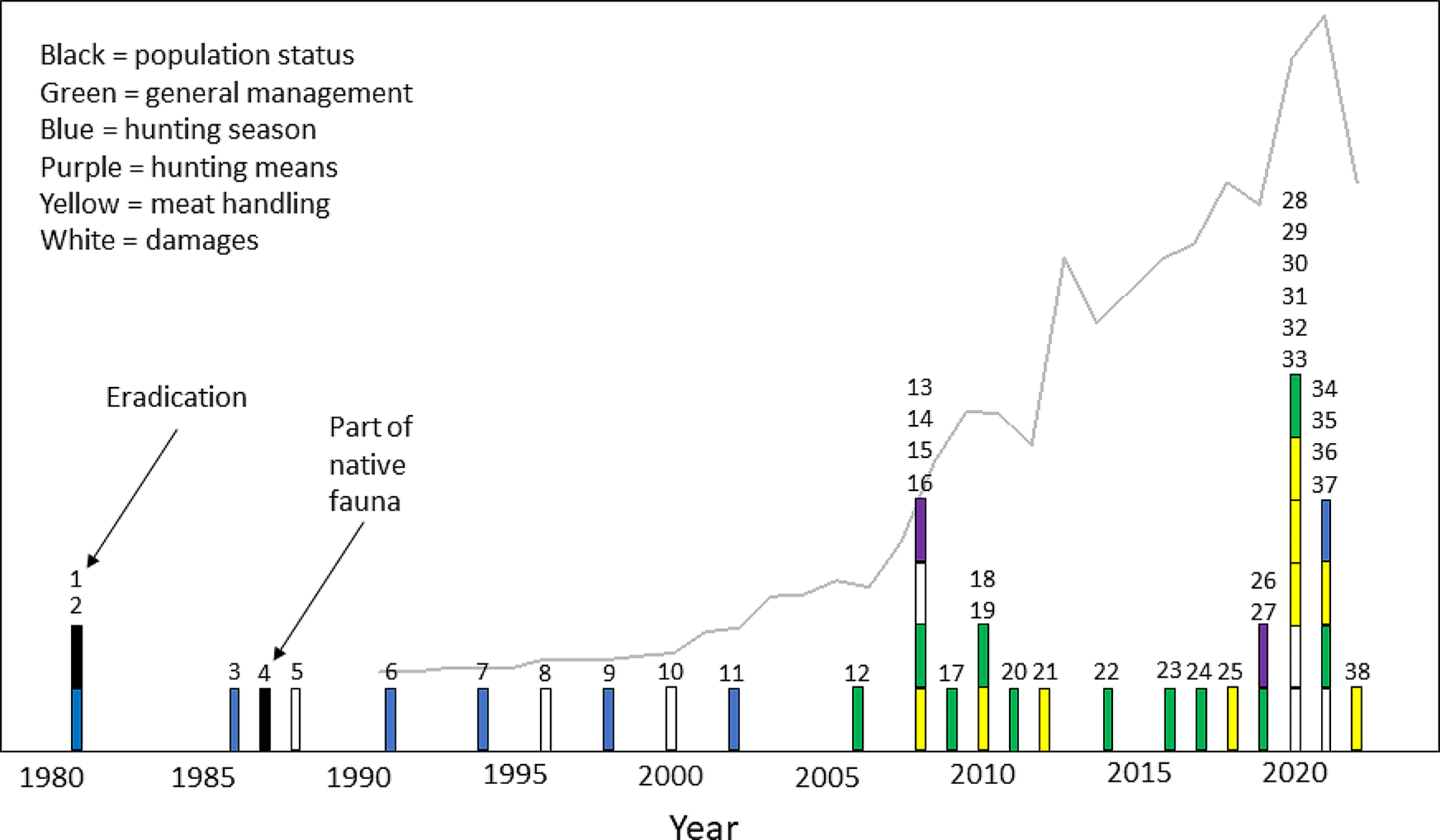
Timeline showing legislative decisions and commissions on wild boar during the period 1981–2022. Two early parliament decisions, marked by arrows, illustrate a key policy shift. First a decision stating that the population should be eradicated, second a decision that wild boar is part of the native fauna and should be allowed in restricted extent. Topics are indicated by bar colours. The estimated harvest of wild boar in Sweden is indicated by the solid grey line. Each number corresponds to a section in Table 1

In total, we recorded 38 legislative decisions and/or commissions regarding wild boar during the period 1981–2022 (Fig. 3; Table 1). The number of decisions increased over time and was on average 0.5 decisions per year for the period 1981–2000, increasing to an average of 1.3 decisions per year for the period 2001–2022. Most decisions in a single year (6) were recorded for 2020.

**Table 1:** Legislative decisions and/or commissions regarding wild boar during 1980–2022

Year	Legislative decision/Commission	SFS number/Registration number	No. in Fig. 3
1981	Eradication of free-living wild boar	SFS 1981:175	1
1981	Females followed by piglets became protected during March - September	SFS 1981:177	2
1986	Protection period for females followed by piglets expanded to January - September	SFS 1985:823	3
1987	Wild boar is part of the native fauna and should be allowed in restricted extent	SFS 1987:259 SFS 1987:905	4
1988	Wild boar included among species allowed for protective hunting all year around if they cause damage to gardens or homesteads	SFS 1988:1175	5
1991	Females followed by piglets became protected all year around	SFS 1991:1770	6
1994	Open season for hunting with dogs specified as August - February	SFS 1994:1454	7
1996	Wild boar included among species allowed for protective hunting all year around if they cause damage to gardens, homesteads or agriculture areas	SFS 1996:727	8
1998	Open season for hunting with dogs changed to August - January	SFS 1998:1000	9
2000	Wild boar included among species where a special game management unit can decide that hunting should be carried out in cooperation to prevent damage	SFS 2000:592	10
2002	Adult wild boar other than females followed by piglets became protected during February 16 – April 15	SFS 2002:551	11
2006	Commission to Swedish EPA: Review literature regarding management and, if needed, suggest measures for durable management	Jo2003/350, 1828; Jo2004/477, 488; Jo2005/1780	12
2008	Allow use of fixed light at places specially arranged for wild boar hunting, e.g., with high stand and bait. CAB can give permission to use thermal images in wild boar hunting	SFS 2008:1412	13
2008	Commission to SBA: Suggest measures to mitigate effects on ecological pig production	Jo2008/3955	14
2008	Commission to Swedish EPA: Develop national management plan for wild boar	Jo2008/3960	15
2008	Commission to SFA: Make impact assessment regarding the possibilities for hunters to sell small amounts of wild boar meat to other consumers, and measures needed for a safe process regarding *Trichinella*	Jo2008/3956; Jo2009/1918	16
2009	Commission to SAHWM: develop recommendations for wild boar hunting and policy for supplementary feeding, develop methods for local/regional monitoring	Jo2008/2619	17
2010	Commission to SAHWM: develop methods for increased cooperation regarding wild boar	Jo2009/2374	18
2010	Commission to SFA: Make impact assessment regarding the possibilities for hunters to sell small amounts of wild boar meat to other consumers, and measures needed for a safe process regarding *Trichinella*	Jo2010/3195	19
2011	Commission to SAHWM: increase knowledge about population densities at local and regional level	Jo2010/1917	20
2012	Commission to SAHWM: increase knowledge about the importance of testing for *Trichinella*	L2011/2148	21
2014	Commission to SAHWM: develop forecasts for population development, counteract inappropriate feeding, propose harvest strategies	L2013/2633/FJS	22
2016	Commission to SAHWM: develop coherent model for feeding, harvest and management	N2015/05189/JFRO	23
2017	Commission to SAHWM: analyse the effects of previous commissions	N2016/06266/JFR	24
2018	Commission to SFA: Make impact assessment regarding the possibilities for hunters to sell small amounts of wild boar meat to other consumers, and measures needed for a safe process regarding *Trichinella*	N2018/04065/DL; N2018/01954/DL	25
2019	Allow use of mobile lights and thermal images in wild boar hunting, thermal lights only allowed in open terrain or at places specially arranged for wild boar hunting	SFS 2019:174	26
2019	Commission to SAHWM: propose strategies and operational measures	N2018/05010/FJR	27
2020	Commission to SBA: Assess the risk and, if needed, suggest preventive actions to impede the introduction and spreading of African Swine Fever in Sweden	N2020/01012/DL; N2019/03259/DL (delvis); N2017/06252/DL (delvis)	28
2020	Commission to SFA: Suggest legislation and develop a model for financial support of system where hunters can sell small amounts of wild boar meat to other consumers	N2020/01010/DL; N2019/03259/DL (delvis); N2017/06252/JL (delvis)	29
2020	Commission to SVA: Elucidate a national digital system for traceability of wild boar meat	N2020/01013/DL; N2019/03259/DL (delvis); N2017/06252/JL (delvis)	30
2020	Commission to CAB in Kronoberg county: Adapt the joint CAB digital system regarding primary producers to also allow for registration of hunters selling wild boar meat, coordinate revision of local management plans for all other CABs with wild boar	N2020/01011/DL; N2019/03259/DL (delvis); N2017/06252/JL (delvis)	31
2020	Commission to Swedish EPA: Review existing models for survey of wild boar damages to agriculture crops	M2020/02056 (delvis); m2020/02000; M2020/01479 m. fl.	32
2020	Commission to SAHWM: propose measures to mitigate damage to agriculture	N2019/02814/FJR	33
2021	Protection period for adult wild boar other than females followed by piglets changed to February - March	SFS 2021:334	34
2021	Introduction of system for subsidised tests of *Trichinella* and Cs-137	SFS 2021:576	35
2021	Give CABs authorization to temporarily prohibit supplementary feeding	SFS 2021:807	36
2021	Commission to Swedish EPA: Develop standardized method for survey of wild boar damage to agriculture crops	M2021/01846; M2021/01186; M2021/00708 m.fl.	37
2022	Commission to SFA: Coordinate communication within the wild boar package together with SVA, Swedish EPA, SBA, CABs	N2022/01539; N2017/06252 (delvis)	38

Swedish EPA = Environmental Protection Agency, SFA = Swedish Food Agency, SBA = Swedish Board of Agriculture, SVA = Swedish Veterinary Agency, CAB = County Administrative Board, SAHWM = Swedish Association for Hunting and Wildlife Management, “delvis” = partly, m.fl. = and others

In the 1980s, the sparse wild boar population located in three core areas was the subject of two parliament decisions, going in opposite directions. The first decision (No. 1 in Fig. 3; Table 1) was that the population should be eradicated with the exception for a sub-population of 50–100 animals residing in the Tullgarn area that should be maintained for research purposes. The Swedish EPA was given the task to monitor the development regarding damage to crops, population growth rate and the possibility to regulate population growth by hunting.

The main result from the monitoring of the Tullgarn population was summarised as (quote from Kristiansson [79], translated from Swedish): “Given that an efficient and well implemented hunting is carried out in combination with measures for damage prevention in agricultural crops, as well as the build-up of an organisation for administration of the wild boar hunting, it should be possible to have wild boar in Sweden.”

Based on the results from the monitoring, as well as a pervasive discussion regarding wild boar as part of the indigenous fauna, the parliament decided in 1987 that wild boar is native to Sweden and should be allowed “in restricted extent”. This second decision implied a key policy shift and entailed that wild boar was included in a major revision of the Hunting Act and Hunting Ordinance (no. 4 in Fig. 3; Table 1).

The additional 36 legislative decisions and/or commissions taken during 1981–2022 concerned various topics. Decisions regarding the hunting season, e.g., the timing of the open season and the use of dogs during that season, dominated in the 1980s and 1990s but comprised only 7 decisions (18%) during the time period as a whole. Most decisions (11, corresponding to 29% of the total) addressed management in general, such as management strategies, monitoring methods or recommendations against inappropriate feeding. Handling of wild boar meat, including testing for *Trichinella* and measures to increase the consumption of wild boar meat, was the subject of 9 decisions (24%), followed by measures to prevent or monitor damage (5 decisions, 13%), including conditional shooting (i.e., the possibility for the person holding hunting rights to protect gardens, homesteads and agricultural areas from damage by shooting animals regardless of rules such as time of the open season or other regulations). Two decisions (5%) each were recorded for population status, damages and hunting means, such as use of fixed or mobile lights or thermal images.

During the time period under study, hunters in Sweden were only allowed to use wild boar meat for personal consumption, or to sell it to a designated game meat handling facility [80]. Most hunters would have a long distance to the nearest designated facility and were therefore unlikely to harvest a larger number of wild boar than that which could be consumed within their household. Consequently, as the wild boar population increased, facilitating harvest of wild boar meat for the market became regarded as a potentially important action to motivate increased harvest, and the Swedish government gave multiple commissions to SFA to evaluate systems where hunters could sell wild boar meat without jeopardising public health safety (nos. 16, 19, 25, 29 in Fig. 3; Table 1). Such a system was already in place in Germany, where trained and certified hunters are allowed to sell wild boar meat to consumers.

In 2019, the parliament also decided to include wild boar within the national food strategy. Among the adopted measures, one was to establish “the wild boar package” with the aim to provide consumers with greater access to, and supply of, wild boar meat as food in a safe way. This decision resulted in three commissions to different government agencies (nos. 30, 31, 38 in Fig. 3; Table 1).

Another food safety issue that concerns wild boar in Sweden is potential cesium (Cs) contamination of the meat. Following the nuclear accident in the Chernobyl nuclear power plant in 1986, some areas in Sweden were contaminated by radioactive fall-out of, mainly, Cs-137. Typical wild boar foraging behaviour involves rooting and consumption of plant material in the soil, and this means that the Cs-137 content can be higher in wild boar meat compared to other mammals of similar size. Therefore, it is possible for Swedish hunters to test their wild boar meat for Cs-137. The cost of testing falls upon the individual hunter, and this has also been perceived as a potential hinderance to reach an increased harvest. Therefore, a system for subsidised tests of *Trichinella* and Cs-137 was introduced in 2021 as a response to the peak in the wild boar population in 2020/2021, but also to the fact that the wild boar population expanded into areas where the fall-out had be large (no. 35 in Fig. 3; Table 1).

### Published research

**Fig. 4:**
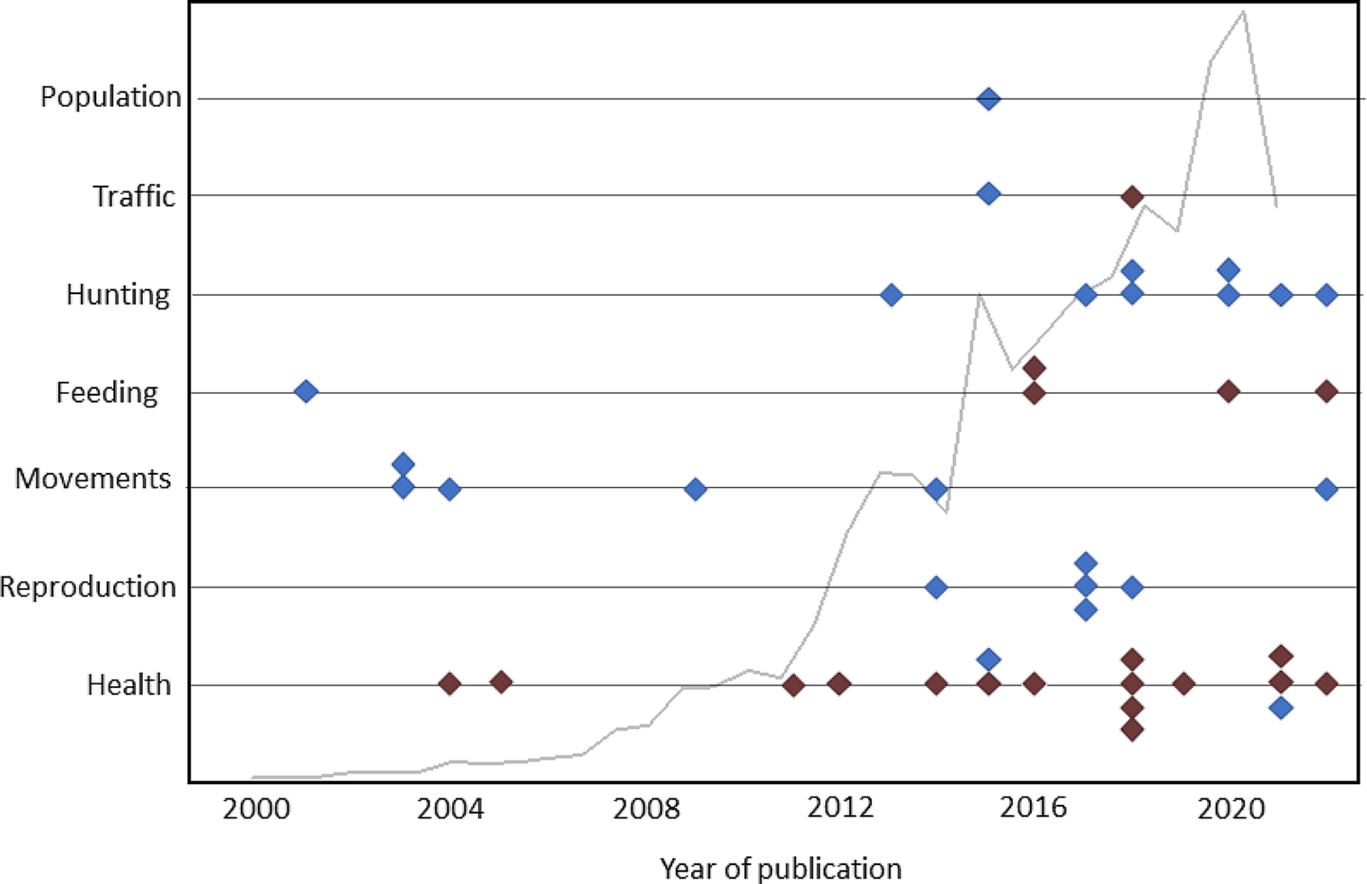
Timeline showing published scientific papers regarding the present free-ranging wild boar population, divided into 7 topics. Each symbol denotes one publication. Red symbols indicate papers focusing on damage, diseases or other adverse effects, blue symbols denote papers describing wild boar ecology and/or management. The estimated harvest of wild boar is indicated by the solid grey line

In total, we found 44 published scientific papers regarding the present free-ranging wild boar population. Publication year ranged from 2001 to 2022 (Fig. 4 and Additional file 3).

The number of publications increased over time. It was on average 0.7 publications per year for the period 2002–2010, increasing to an average of 3.1 publications per year for the period 2011–2022. The highest number of publications in a single year (8) were recorded in 2018.

Health including diseases was the most common topic and included 17 publications (39% of the total), followed by hunting (8: 18%) and movements including natal dispersal and home ranges (6: 14%). Five publications (11%) each were recorded for reproduction and feeding, the latter including rooting and damages. Two publications (5%) were recorded for traffic and one publication (2%) for population development. Studies focusing on damage, diseases or other adverse effects of wild boar were recorded for three topics: health (15 of 17), feeding (4 of 5) and traffic (1 of 2).

In general, the earliest topics to be subject to research studies were related to basic ecologic features such as movements and feeding patterns, whereas later research focuses more on hunting, reproduction, and population development. One interesting example of an expansion in research focus is the paper by von Essen [81], here included in the group “Hunting”, where wild boar hunting is discussed from an ethical standpoint.

In addition to the studies mentioned above, we found three large-scale studies conducted at the European or Eurasian level, that includes Swedish data: Keuling et al. [82] regarding mortality rates of wild boar, Massei et al. [83] regarding the development of wild boar populations and the number of hunters, and Markov et al. [23] regarding the northern distribution limit of wild boar in Eurasia. Data on the Swedish wild boar population is also included in the works of the Enetwild group [84], a project run by EFSA and aiming at modelling species distribution and abundance of selected host species and their pathogens.

## Discussion

According to the current strategy of the Swedish EPA, game management in Sweden should balance three overall perspectives that are equally important [85]. The first perspective is the conservation of viable populations of all native species and their environment, acknowledging the intrinsic value of species in addition to other values, such as ecosystem services. The second perspective is that game species is a resource that should be used wisely. Hunting is one form of use but so is, for instance, birdwatching. The third perspective is the necessity to limit damage and other inconveniences from game. In this context, it is specifically mentioned that landowners and hunters, in accordance with the Swedish Hunting Act, has a shared responsibility to do so. In our discussion, we analyse and discuss the development of the Swedish wild boar population, and the legislative decisions taken, in relation to these perspectives.

### Perspective 1 – conservation of viable populations of native species

Historically, and before our study period, the aim of the state was to maintain the status of wild boar as extinct. Thus, wild boar was clearly regarded as a pest species. In line with this view, the first political decision during our study period (in 1981) was to eradicate the free-ranging wild boar population, except for a small sub-population that was to be retained for research purposes. However, this decision was immediately followed by a decision to protect females followed by piglets during the time of the year when piglets are most common, i.e., during the wild boar parturition peak. This is in line with established hunting ethics where females with dependent young should not be killed as this indirectly leads to unnecessary suffering and the death of the young. Protection of huntable species during the rearing of the young and various stages of reproduction is also a prerequisite for sustainable hunting according to the Birds Directive of the EU. For wild boar in Sweden, this period of protection was later further expanded. However, it should be noted that the piglets remain open for hunting year around and, once the piglets are harvested, the female is no longer followed by piglets and can be harvested as well. Hence, the decision in 1981 to eradicate the free-ranging wild boar population but also protect females followed by piglets can be interpreted as a wish to “eradicate while upholding ethical principles” rather than “eradicate at all costs”. The responsibility to control the sub-population that should be kept for research purposes at a low level, and to eradicate wild boar in other areas, were given to the respective County Administrative Boards (CAB). However, we have not registered any specific measures taken to enforce this decision, and to our knowledge no such measures were taken. We therefore assume that this was rather left to potential voluntary efforts by hunters, who may have been reluctant to do this. At that time, when there were only three small sub-populations of wild boar in Sweden, there was no tradition of wild boar hunting and decisions were largely based on experiences from other countries [86].

In 1987, a key political decision regarding the free-ranging wild boar population went in the opposite direction, acknowledging wild boar as part of the native fauna with residence rights. In the eyes of the state, the view of wild boar thus changed from a pest to be eradicated to a native species to be the subject of conservation and management. Notably, the Convention on Biological Diversity was negotiated and opened for signature a few years later, in 1992 [87]. Hence, we suggest that the political decision to allow wild boar in Sweden may be an early example of a more contemporary view on the responsibility to preserve species. The decision was partly based on archaeological findings showing that wild boar was part of the native fauna [44], but also on the monitoring of the sub-population by the Swedish EPA. Parliament protocols show that the discussion was harsh, with strong advocates on both sides, where hunting and conservation interests wanted to allow wild boar, whilst farming interests wanted to keep wild boar extinct. The resulting decision to allow wild boar in restricted extent was probably a compromise. We have not found any definition of the term “restricted extent”. It appears obvious, however, that the size of the present wild boar population exceeds what was regarded as restricted extent at the time of the decision.

### Perspective 2 – wise use of game as a resource

The second cornerstone of the Swedish EPA´s strategy for game management is to promote the use of game as a resource for different purposes, including both cultural ecosystem services such as wildlife watching, tourism and the cultural aspects of hunting, and provisioning ecosystem services such as meat and other products. Wise use requires sustainable harvest of game species, and the Swedish EPA points out that also ethical perspectives should be accounted for [11, 85].

As the wild boar population grew, the number of political decisions increased, although slowly at first, and commissions to government agencies became more common. Research on Swedish wild boar also started, which should reflect knowledge needs associated with the conservation and management of a recolonising species. The first topics to be covered included basic ecological features such as home ranges and foraging. Later research also covered topics such as diseases, hunting and traffic accidents, which could reflect new knowledge needs with an increasing population. So far, we have only registered one paper modelling the size of the wild boar population [88], but we anticipate more such studies in the future. In some cases, legislative decisions have been directly informed by results from research studies. For instance, a decision in 2021 to bring forward in time the closed season when all adult wild boars are protected from hunting, from February 16 – April 15 to February 1 – March 31 (no. 34 in Fig. 3; Table 1), was based on findings showing that the main reproductive season occurred somewhat earlier than previously known [20]. The intention was to further minimise the risk of accidently harvesting females with piglets during peak parturition when piglets to a larger extent may be left in the nest and consequently do not follow the female and are not visible for the hunter.

Wild boar can sporadically be infected by *Trichinella* parasites or contaminated by salmonella, and these zoonoses can be transferred to humans through undercooked meat. The risk that the meat has not been properly tested for *Trichinella* is one reason why hunters are only allowed to sell wild boar meat to designated facilities that are required to test the meat before it reaches the market. Although public health and food quality is a concern, these restrictions in provisioning wild boar meat for the market has been regarded as an obstacle, preventing increased harvest. To hunt wild boar without consuming the meat does not comply with wise use of the resource. The Swedish government has therefore been eager to develop a system where individual hunters can sell wild boar meat to end consumers in a secure way, resulting in several commissions, and later the implementation of the so called “wild boar package”, designed to increase the access to wild boar meat for the market. As can be noted in Table 1, four commissions to SFA are very similarly worded, providing an interesting example of the, sometimes, intricate interplay between a government and a government agency. When SFA received the first commission regarding this matter in 2008 (no. 16 in Fig. 3; Table 1), the agency failed to find a system which could be regarded as sufficient from a food safety perspective. The second commission in 2010 (no. 19 in Fig. 3; Table 1) proposed a system that was deemed too complicated and expensive. The third commission in 2018 (no. 25 in Fig. 3; Table 1) was regarded as acceptable but lacking in detail. The fourth commission (no. 29 in Fig. 3; Table 1) was a directive to suggest legislation and develop a model for financial support of a system where hunters can sell wild boar meat in a safe way, including, e.g., a requirement for hunters to pass a course on testing procedures, hygiene, and meat traceability to be allowed to sell wild boar meat to end consumers. Although SFA has completed this commission, such a system is still not in place as this is written.

In the annual commission to SAHWM, which is responsible for parts of the wildlife management in Sweden, wild boar is first mentioned in 2009 and has been included ever since. Implemented measures regarding wild boar includes information about efficient and ethical hunting methods, but also, e.g., proper procedures for supplementary feeding and the importance of testing wild boar meat for *Trichinella*.

However, the wild boar population is presently decreasing whereas a system for hunters to sell wild boar meat, designed both to increase the harvest and the availability of wild boar meat at the market, is still not in place. Such time-lags and temporal mismatch in political decisions are not uncommon and can be found also for other species than wild boar. For instance, the barnacle goose (*Branta leucopsis*) is legally protected in the EU according to the Birds Directive, although the species has increased towards superabundance. It is politically very difficult to change the status, which presently makes it impossible to set goals for population reduction. At the same time, hunting remains open for much less abundant goose species [89]. Another example of management mismatch is reported by Swenson et al. [90], who evaluated whether the actual brown bear (*Ursus arctos*) population development in Sweden correspond to management-decided national objectives during five management regimes between 1943 and 2013 and found that the objectives were only met in one of the periods.

### Perspective 3 – limitation of damage

The third cornerstone of the Swedish EPA´s strategy for game management is to limit damages caused by game species. These may comprise negative effects on the ecosystem of overabundant species or negative effects on socio-economic interests and land use. For wildlife with an open season, hunting is considered the main method to regulate, limit and control the size of game populations and hence reduce damages that are related to the density of wildlife populations [85].

Overall, our analysis show that wild boar population growth can be rapid and exponential during a recolonisation phase. The growth pattern, as manifested in estimated harvest and traffic accidents, suggests that the wild boar population in Sweden grew exponentially until around 2010/2011, but then levelled off, and that logistic models provided a significantly better fit for both data series over the whole study period. Although the analysis does not explicitly reveal what process caused this change, a shift to logistic growth indicates that a density dependent mechanism has come into play. The wild boar population could, e.g., be approaching its ecological and/or cultural carrying capacity. The latter would imply that the population size tolerated by society has been reached, resulting in management actions such as increased hunting pressure in response to increased population density, potentially regulating the population. As for the ecological carrying capacity, it should be noted that more than 20% of the hunting teams in the counties under study reported that wild boar populations were not established during 2020/2021, and an additional 30% reported only sporadic occurrence of wild boar. This suggests that suitable habitats are still available and that the ecological carrying capacity has not yet been reached.

In contrast to estimated harvest, for which logistic growth models consistently showed a better fit for the time series that incorporated years after 2013/2014, the number of traffic accidents temporarily shifted back to exponential growth for time series including 2015/2016 to 2019/2020. We cannot conclusively determine the reason for this discrepancy. However, wild boars are increasingly inhabiting urban areas [91] where traffic is more intense, and it is possible that this is reflected in the number of traffic accidents. Also, we cannot rule out that the decrease in traffic accidents during the following period, 2019/2020 to 2021/2022, is partly due to reduced traffic during the covid pandemic [92]. However, both estimated harvest and the number of traffic accidents decreased in the last 2–3 years of our time series. As Sweden never had a strict lock-down and all data sets show similar trends, we suggest that the decline in number of traffic accidents rather reflects an actual decline in population size. Either way, the decline in traffic accidents contributes to support the shift back to logistic growth for the time series that include the last years. A potential alternative explanation for the decline in wild boar population size at this time is a decision by the government in 2019 to allow the use of movable lights and thermal images in wild boar hunting (no. 26 in Fig. 3; Table 1). Most wild boars are harvested during night-time at feeding stations or on agricultural fields [37] and, according to anecdotal reports from hunters, this has greatly increased hunting efficiency. We suggest this government decision to be a main reason for the increase in harvest, and subsequent decrease in population size, during the last years of our study. However, in the case of the latter, other contributing factors could be adverse weather conditions during the main reproductive season or diseases.

In this study, we only attempted to fit basic exponential and logistic models to wild boar data as our time series are relatively short, covering only the initial recolonisation phase. With longer time series, it could be informative to also evaluate additional models, e.g., where the population reach an asymptote to then decline. The future will have to tell if the present decline in the Swedish wild boar population will be short-term or sustained, and/or if the wild boar population will start to fluctuate around its ecological or cultural carrying capacity as could be expected given a logistic growth pattern.

In many countries, including Sweden, game management largely relies on recreational hunting. Over time, the number of hunters has decreased in many European countries, and this has raised questions about the ability of recreational hunters to maintain sufficient harvest levels to reach management goals [83]. This question is particularly relevant regarding wild boar, as studies have shown that the current harvest levels generally fall short of the species reproductive output [82]. Based on our study, and in agreement with results by [93], we conclude that it is possible to reach sufficient harvest levels in recreational hunting if the hunters receive proper training and that legislation allows for efficient equipment such as thermal images.

Nevertheless, the wild boar population in Sweden grew rapidly during large parts of our study period, its spatial expansion is likely to continue, and the population may well start to increase again in response to, e.g., a reduced harvest. Thus, there is a continuous need for measures to keep up or increase the harvest and thereby limit damages. The wild boar is already subject to the most generous open hunting season of all ungulates in Sweden and, when open, allows hunting 24 h a day. Although females followed by piglets are protected year-around, juvenile wild boar are allowed for hunting year-around and adult wild boar have only a short protection period during peak parturition. Hence, there are few possibilities to further increase the open hunting season without increasing the risk of unethical hunting where adult females with dependent young are killed. In addition, a variety of hunting methods are allowed, such as still hunting (including use of bait and light), drive hunts with or without dogs, hunting with dogs that bark at bay, stalking and live traps [37]. The use of dogs, however, is restricted to the autumn and winter period. A total of ten different types of live traps are allowed for wild boar, but only for juveniles [94]. The reason is to avoid that a female is caught while her piglets are left outside the trap. Even so, the use of live traps is very limited [37], as this method is deemed unethical by many Swedish hunters.

As part of its government assignment, SAHWM has developed several activities to help mitigate crop damage. On example is “the wild boar barometer”, an expert system where SAHWM personnel on a monthly basis advice farmers and hunters regarding the risk of wild boar damage to agricultural crops in their respective counties and the associated need to adjust the hunting pressure. The risk assessment is based on the current state of the wild boar population, e.g., related to wild boar reproductive success which fluctuates between years due to weather conditions around the time of peak parturition as well as food availability (acorn mast years), and the wild boar barometer provides a “stop-sign” classification for each county: green (low risk), yellow (medium risk) and red (high risk) [95]. Another example is “the wild boar night”, where SAHWM organise local hunters to joint efforts during appointed nights. Participating hunters hunt where they already hold hunting rights and post in the vicinity of agricultural fields with vulnerable crops. When a wild boar is harvested the remaining animals in the group usually run away and does not provide an opportunity to simultaneously harvest another wild boar in the same field. They often, however, move to another field close by and may give the opportunity for additional harvest there, hence increasing the total harvest and making wild boar hunting and crop protection more efficient. Harvested wild boars are either kept by the hunter/landowner or given to the municipality who distributes the meat to schools and retirement homes. Each participating hunter (around 250 hunters on each occasion) are awarded a lottery ticket with a chance to win hunting equipment, and around 40 wild boars have been harvested on each occasion. In addition to increased harvest and protection of agricultural crops, one aim is to stimulate co-operation between hunters and farmers.

It is clear that the historic conflict regarding wild boar damages has resurfaced following the return of wild boar. The opinion that wild boar should be re-eradicated in Sweden remains [96] and is regularly expressed in the public debate. At present however, the main strategy of the state to handle this human-wildlife interaction is to be adaptive to changing conditions. The aim is to achieve a balance between the conservation of a viable population where wild boar also is considered a resource that should be used wisely, and wild boar damages through population regulation and limitation. Nevertheless, when it comes to wild boar, it is also relevant to ask if there are situations when the human-wildlife interaction again would require more drastic management strategies.

#### Limitation of damage – wild boar extirpation to combat ASF

The prerequisites for wild boar management in Sweden changed on September 6, 2023. At that time, Sweden detected its first case of ASF [97], a viral disease that has spread in Eurasia since 2007, and within the EU since 2014. ASF poses a severe threat to free-ranging wild boar populations, animal disease control, domestic pig production and the pork industry [32, 33, 98]. The geographical location of Sweden, demarked by the Baltic Sea towards mainland Europe, acts as a barrier against natural introduction, i.e., direct virus transmission through animal contact and movement within and between wild boar populations [97]. However, the virus also has the potential for indirect transmission through infected carcasses and contaminated environments, as well as for long-distance spread through anthropogenic movements of, e.g., animals, contaminated feed and pork products that cause point source incursions in new areas [32, 33, 98]. The outbreak in Sweden has consequently been attributed to a long-distance leap caused by anthropogenic activities, most likely non-commercial food import [97].

The outbreak in Sweden is managed according to disease control measures for ASF decided by the EU, which aims to eradicate the virus in areas where point incursions occur. Viral eradication requires actions such as zoning and delimitation of a restriction area where the virus is present, fencing of the area, search for and destruction of wild boar carcasses as these are a source of environmental disease transmission, extirpation of the wild boar population through killing with hunting-like methods according to the Epizootic Act (SFS 1999:657), and intensified hunting outside the area to reduce wild boar population density and the risk of direct transmission to other areas [98, 99]. The actions to eradicate the virus involve Swedish government agencies as well as SAHWM and voluntary efforts by local hunters. SBA has the main responsibility both for national plans to prevent ASF entering Sweden and, in case of outbreaks, decisions on restrictions and actions (no. 28 in Fig. 3; Table 1). SVA is the authority responsible for disease monitoring, both to detect potential outbreaks and during outbreaks. SAHWM has provided knowledge of wild boar in general as well as local knowledge on wild boar in the outbreak area. The Association has also been responsible for the organisation of local hunters who participate in carcass searches and wild boar extirpation through hunting-like methods. Such actions continue as part of the ongoing monitoring.

By December 2023, analyses of wild boar carcasses found through searches and wild boars killed with hunting-like methods suggested that the virus had not spread outside a relatively small core area of 21 km^2^. The analyses also showed that the last death from ASF occurred in late September, and that there is currently no active disease spread in the area [97]. In total, 63 wild boars were infected out of 162 found dead or killed with hunting-like methods. If virus eradication through extirpation of wild boar in the restriction area succeeds, wild boar will eventually be able to recolonise from surrounding areas. Thus, the actions against ASF would have stopped further viral spread, eventually throughout Sweden, and saved the free-ranging wild boar population from long-term suffering and substantial reduction due to disease. Successful management of the disease may also have been a prerequisite for future human-wildlife co-existence in Sweden.

## Conclusions

Based on our study, we suggest that all three perspectives established by the Swedish EPA in the national game management strategy are accounted for in Swedish wild boar management: conservation, use of wild boar as a resource, and limitation of damages. However, the relative importance of the different perspectives has shifted over time. Historically and initially during our study period, wild boar was regarded as a pest in the eyes of the state and the aim was to eradicate the free-ranging population. Since the political decision in 1987, establishing that wild boar is part of the native fauna, the wild boar population has increased substantially. Although wild boar now is present in most of southern Sweden, areas remain where wild boar is absent. Thus, the wild boar population is likely to continue to expand until all suitable habitats are occupied, unless actively stopped. Wild boar population growth has shifted the focus of the state from wild boar conservation to sustainable management and limitation of damage. An exception is the very recent outbreak of ASF, which necessitates the stamping out strategy recommended by the EU, where the aim is to eradicate the virus through extirpation of the local wild boar population [99].

It is also obvious that ethical and other animal welfare considerations are important in Swedish wildlife management and hunting legislation. One example is that wild boar females followed by piglets are protected year-around, and that this decision was taken whilst the state goal was to eradicate the free-ranging population. Other examples can be found in the timing of the open hunting season for adult wild boars other that females followed by piglets and the fact that live traps cannot be designed so that adult wild boars may be caught, further minimising the risk for piglets to be subject to unnecessary suffering.

However, we also conclude that it has become increasingly important over time to find ways to slow down wild boar population growth, or even reduce the population, especially during the latter part of our study period. Increased use of wild boar meat as a resource is regarded as an important way to increase the total harvest and reduce damage, in accordance with the strategy of the Swedish EPA. The wise use perspective is also manifested in decisions such as the wild boar package. Creating a system where hunters can sell wild boar meat in a safe way from a public health perspective has for several years been regarded by the state as a main measure in order to increase the harvest. However, at the time of writing, such a system is still not in place, something we interpret as a temporal mismatch in the decision process. We suggest that the political decision to allow the use of movable lights and thermal images has increased the efficiency of night hunting and contributed to wild boar population control, as a population decline is likely reflected both in harvest and traffic accident data. Hence, we conclude that recreational hunting can control a wild boar population given the proper prerequisites.

Finally, the prerequisites for management of the Swedish wild boar population have been changed with the outbreak of ASF. It provides a topical example of how unexpected events can induce a necessary shift in management strategy, from sustainable management to extirpation, and where successful stamping out actions against the virus should be followed by a new period where a conservation strategy is applied as wild boar can be allowed to recolonise the area after it has been confirmed disease free. This illustrates the need for adaptive management as human-wildlife interactions and trade-offs constantly change.

### Electronic supplementary material

Below is the link to the electronic supplementary material.

Supplementary Material 1. Additional file 1. ANOVA tables for analyses of growth pattern (Word file).

Supplementary Material 2. Additional file 2. Swedish Code of Statue referenced in the text (Word file).

Supplementary Material 3. Additional file 3. Published scientific papers found in literature search (Word file).

## Data Availability

All harvest estimates are publicly available at https://rapport.viltdata.se/statistik/ All data on traffic accidents are publicly available at https://viltolycka.se/statistik/ Data on establishment of wild boar is available from the corresponding author on reasonable request.Data on *Trichinella* tests are publicly available in annual reports at https://sva.se/vilda-djur/arsrapporter-sjukdomsovervakning-vilda-djur/ Swedish Code of Statue for the period June 9, 1988, to March 29, 2018, are publicly available at http://rkrattsdb.gov.se/sfspdf Swedish Code of Statue for the period March 30, 2018, until present is publicly available at http://svenskforfattningssamling.se Swedish Code of Statue for the period previous to June 9, 1988, is publicly available at the library of the Swedish parliament.

## Abbreviations

ANOVA: Analysis of Variance
ASF: African Swine Fever
CAB: County Administrative Board (Swedish: Länsstyrelsen)
EFSA: European Food Safety Agency
EU: European Union
HMP: Hunting Management Precinct (Swedish: Jaktvårdskrets)
NGO: Non-Government Organisation
SBA: Swedish Board of Agriculture (Swedish: Jordbruksverket)
SAHWM: Swedish Association for Hunting and Wildlife Management (Swedish: Svenska Jägareförbundet)
SFA: Swedish Food Agency (Swedish: Livsmedelsverket)
SFS: Swedish Code of Statue (Swedish: Svensk Författnings Samling)
SVA: Swedish Veterinary Agency (Swedish: Statens Veterinärmedicinska Anstalt)
Swedish EPA: Environmental Protection Agency (Swedish: Naturvårdsverket)
