# Supplementary material for: From virtually extinct to superabundant in 35 years: establishment, population growth and shifts in management focus of the Swedish wild boar (Sus scrofa) population
Source: BMC Zool. 2024 Jul 1;9:14. doi: 10.1186/s40850-024-00202-2 (PMC11218266; doi:10.1186/s40850-024-00202-2)
Supplement: Supplementary file 1 — Supplementary Material 1. Additional file 1. ANOVA tables for analyses of growth pattern (Word file). [file 40850_2024_202_MOESM1_ESM.docx]

Additional file 1

Parameter values for the exponential and logistic models fitted by nls, following (Stevens MHH. A primer in Ecology with R. Springer Science, New York. 2009. <https://doi.org/10.1007/978-0-387-89822-7>) for the time series *estimated harvest* and *traffic accidents* as a whole (1990/1991 to-2021/2022 and 2003/2004 to 2021/2022, respectively), and for each time series stepwise shortened by one hunting year. The equations used for exponential and logistic growth are shown in Methods (Statistical analyses). ANOVA was applied to test if the logistic model provided a significantly better fit to the data. If there is no significant difference, the exponential (parsimonious) model is considered the best model. Likewise, if the best logistic model fitted by nls is not ecologically realistic, as defined by a negative *α*, the exponential model is considered the best model as the smallest ecologically realistic alpha value (*α* = 0) implies that nls would fit a model with parameter values identical to the exponential model. Exp = exponential model, Log = logistic model, NA = not applicable, years = hunting years (July 1 – June 30) starting in the specified year, e.g., 1990 denotes the hunting year 1990/1991 (July 1 1990 to June 30 1991). Model fitting with nls and tests were performed in R 3.6.3 (R Core Team. R: a language and environment for statistical computing. Vienna, Austria. 2020. <https://www.R-project.org/>).

| **Fitted model** | **Estimated harvest** | | | | | | | | |  | **Traffic accidents** | | | | | | | | |
| --- | --- | --- | --- | --- | --- | --- | --- | --- | --- | --- | --- | --- | --- | --- | --- | --- | --- | --- | --- |
|  | **Years (*N*)** | **Parameter values** | | | **ANOVA** | | | | **Best model** | | **Years (*N*)** | **Parameter values** | | | **ANOVA** | | | | **Best model** |
|  |  | *N*_0_ | *r* | *α* | Res.df | Res.Sum.sq | *F* | Pr (>F) |  |  |  | *N*_0_ | *r* | *α* | Res.df | Res.Sum.sq | *F* | Pr (>F) |  |
| Exp | 1990-2021 | 6263 | 0.10 | NA | 30 | 6675108309 |  |  |  |  | 2003-2021 | 1336 | 0.091 | NA | 17 | 14449662 |  |  |  |
| Log | (*N* = 32) | 573 | 0.25 | 0.0000065 | 29 | 2666623514 | 43.59 | < 0.0001 | Log |  | (*N* = 19) | 561 | 0.25 | 0.00013 | 16 | 7984224 | 12.96 | 0.0024 | Log |
| Exp | 1990-2020 | 4711 | 0.12 | NA | 29 | 3507499824 |  |  |  |  | 2003-2020 | 1113 | 0.11 | NA | 16 | 6461561 |  |  |  |
| Log | (*N* = 31) | 1034 | 0.21 | 0.0000053 | 28 | 1988588625 | 21.39 | < 0.0001 | Log |  | (*N* = 18) | 69 | 0.20 | 0.000097 | 15 | 4540972 | 6.34 | 0.024 | Log |
| Exp | 1990-2019 | 4461 | 0.12 | NA | 28 | 3426888053 |  |  |  |  | 2003-2019 | 960 | 0.12 | NA | 15 | 3392561 |  |  |  |
| Log | (*N* = 30) | 613 | 0.24 | 0.0000065 | 27 | 1647898739 | 29.15 | < 0.0001 | Log |  | (*N* = 17) | 823 | 0.15 | 0.000047 | 14 | 3200143 | 0.84 | 0.37 | Exp |
| Exp | 1990-2018 | 4362 | 0.12 | NA | 27 | 3414654182 |  |  |  |  | 2003-2018 | 894 | 0.13 | NA |  |  |  |  |  |
| Log | (*N* = 29) | 275 | 0.29 | 0.0000078 | 26 | 1045805955 | 58.89 | < 0.0001 | Log |  | (*N* = 16) | 911 | 0.13 | −0.0000095 | Log model ecologically unrealistic | | | | Exp |
| Exp | 1990-2017 | 3551 | 0.13 | NA | 26 | 2615023699 |  |  |  |  | 2003-2017 | 957 | 0.12 | NA | 13 | 2569323 |  |  |  |
| Log | (*N* = 28) | 2958 | 0.29 | 0.0000077 | 25 | 1041916822 | 37.74 | < 0.0001 | Log |  | (*N* = 15) | 726 | 0.20 | 0.00011 | 12 | 2216548 | 1.91 | 0.19 | Exp |
| Exp | 1990-2016 | 3109 | 0.14 | NA | 25 | 2377446354 |  |  |  |  | 2003-2016 | 942 | 0.12 | NA | 12 | 2551819 |  |  |  |
| Log | (*N* = 27) | 208 | 0.31 | 0.0000084 | 24 | 971066015 | 34.76 | < 0.0001 | Log |  | (*N* = 14) | 625 | 0.24 | 0.00015 | 11 | 2033898 | 2.80 | 0.12 | Exp |
| Exp | 1990-2015 | 2485 | 0.15 | NA | 24 | 1899105848 |  |  |  |  | 2003-2015 | 967 | 0.12 | NA | 11 | 2507742 |  |  |  |
| Log | (*N* = 26) | 215 | 0.31 | 0.0000083 | 23 | 970585690 | 22.00 | 0.00010 | Log |  | (*N* = 13) | 430 | 0.36 | 0.00022 | 10 | 1160869 | 11.60 | 0.0067 | Exp |
| Exp | 1990-2014 | 1973 | 0.16 | NA | 23 | 1569159512 |  |  |  |  | 2003-2014 | 915 | 0.13 | NA | 10 | 2330280 |  |  |  |
| Log | (*N* = 25) | 217 | 0.31 | 0.0000083 | 22 | 970556787 | 13.57 | 0.0013 | Log |  | (*N* = 12) | 377 | 0.41 | 0.00024 | 9 | 1057228 | 10.84 | 0.0093 | Log |
| Exp | 1990-2013 | 1463 | 0.18 | NA | 22 | 1228433183 |  |  |  |  | 2003-2013 | 820 | 0.15 | NA | 9 | 1844147 |  |  |  |
| Log | (*N* = 24) | 279 | 0.29 | 0.0000075 | 21 | 953620708 | 6.05 | 0.023 | Log |  | (*N* = 11) | 382 | 0.40 | 0.00024 | 8 | 1056545 | 5.96 | 0.040 | Log |
| Exp | 1990-2012 | 942 | 0.20 | NA | 21 | 844054414 |  |  |  |  | 2003-2012 | 686 | 0.18 | NA | 8 | 1069209 |  |  |  |
| Log | (*N* = 23) | 635 | 0.23 | 0.0000028 | 20 | 831076474 | 0.31 | 0.58 | Exp |  | (*N* = 10) | 482 | 0.31 | 0.00018 | 7 | 936693 | 0.99 | 0.35 | Exp |
| Exp | 1990-2011 | 1274 | 0.18 | NA | 20 | 709301338 |  |  |  |  | 2003-2011 | 713 | 0.17 | NA | 7 | 1043568 |  |  |  |
| Log | (*N* = 22) | 115 | 0.36 | 0.000012 | 19 | 440837225 | 11.57 | 0.0030 | Log |  | (*N* = 9) | 274 | 0.54 | 0.00031 | 6 | 496369 | 6.61 | 0.042 | Log |
| Exp | 1990-2010 | 650 | 0.22 | NA | 19 | 253686751 |  |  |  |  | 2003-2010 | 650 | 0.19 | NA | 6 | 928395 |  |  |  |
| Log | (*N* = 21) | 340 | 0.27 | 0.0000050 | 18 | 235383886 | 1.40 | 0.25 | Exp |  | (*N* = 8) | 255 | 0.58 | 0.000325 | 5 | 482988 | 4.61 | 0.085 | Exp |
| **Fitted model** | **Estimated harvest (*continued*)** | | | | | | | | |  |  |  |  |  |  |  |  |  |  |
|  | **Years (*N*)** | **Parameter values** | | | **ANOVA** | | | | **Best model** | |  |  |  |  |  |  |  |  |  |
|  |  | *N*_0_ | *r* | *α* | Res.df | Res.Sum.sq | *F* | Pr (>F) |  |  |  |  |  |  |  |  |  |  |  |
| Exp | 1990-2009 | 375 | 0.26 | NA |  |  |  |  |  |  |  |  |  |  |  |  |  |  |  |
| Log | (*N* = 20) | 827 | 0.19 | −0.000012 | Log model ecologically unrealistic | | | | Exp |  |  |  |  |  |  |  |  |  |  |
| Exp | 1990-2008 | 461 | 0.24 | NA |  |  |  |  |  |  |  |  |  |  |  |  |  |  |  |
| Log | (*N* = 19) | 1083 | 0.16 | −0.000026 | Log model ecologically unrealistic | | | | Exp |  |  |  |  |  |  |  |  |  |  |
| Exp | 1990-2007 | 805 | 0.20 | NA | 16 | 71293122 |  |  |  |  |  |  |  |  |  |  |  |  |  |
| Log | (*N* = 18) | 253 | 0.32 | 0.000022 | 15 | 55510641 | 4.26 | 0.057 | Exp |  |  |  |  |  |  |  |  |  |  |
| Exp | 1990-2006 | 837 | 0.20 | NA | 15 | 70861023 |  |  |  |  |  |  |  |  |  |  |  |  |  |
| Log | (*N* = 17) | 61 | 0.47 | 0.000037 | 14 | 31591480 | 17.40 | 0.00094 | Log |  |  |  |  |  |  |  |  |  |  |
| Exp | 1990-2005 | 504 | 0.24 | NA | 14 | 28266917 |  |  |  |  |  |  |  |  |  |  |  |  |  |
| Log | (*N* = 16) | 161 | 0.36 | 0.000025 | 13 | 21430615 | 4.15 | 0.063 | Exp |  |  |  |  |  |  |  |  |  |  |
| Exp | 1990-2004 | 362 | 0.27 | NA | 13 | 20657816 |  |  |  |  |  |  |  |  |  |  |  |  |  |
| Log | (*N* = 15) | 236 | 0.32 | 0.000013 | 12 | 19884163 | 0.47 | 0.51 | Exp |  |  |  |  |  |  |  |  |  |  |
| Exp | 1990-2003 | 210 | 0.32 | NA |  |  |  |  |  |  |  |  |  |  |  |  |  |  |  |
| Log | (*N* = 14) | 609 | 0.18 | −0.000097 | Log model ecologically unrealistic | | | | Exp |  |  |  |  |  |  |  |  |  |  |
| Exp | 1990-2002 | 371 | 0.26 | NA |  |  |  |  |  |  |  |  |  |  |  |  |  |  |  |
| Log | (*N* = 13) | 507 | 0.21 | −0.000042 | Log model ecologically unrealistic | | | | Exp |  |  |  |  |  |  |  |  |  |  |
| Exp | 1990-2001 | 341 | 0.27 | NA |  |  |  |  |  |  |  |  |  |  |  |  |  |  |  |
| Log | (*N* = 12) | 822 | 0.076 | −0.00066 | Log model ecologically unrealistic | | | | Exp |  |  |  |  |  |  |  |  |  |  |
| Exp | 1990-2000 | 626 | 0.20 | NA | 9 | 2502007 |  |  |  |  |  |  |  |  |  |  |  |  |  |
| Log | (n = 11) | 271 | 0.42 | 0.00016 | 8 | 1583757 | 4.64 | 0.063 | Exp |  |  |  |  |  |  |  |  |  |  |
| Exp | 1990-1999 | 563 | 0.21 | NA | 8 | 2273182 |  |  |  |  |  |  |  |  |  |  |  |  |  |
| Log | (*N* = 10) | 217 | 0.48 | 0.00019 | 7 | 1481316 | 3.74 | 0.094 | Exp |  |  |  |  |  |  |  |  |  |  |
| Exp | 1990-1998 | 499 | 0.23 | NA | 7 | 2074673 |  |  |  |  |  |  |  |  |  |  |  |  |  |
| Log | (*N* = 9) | 184 | 0.53 | 0.00022 | 6 | 1424091 | 2.74 | 0.15 | Exp |  |  |  |  |  |  |  |  |  |  |
| Exp | 1990-1997 | 370 | 0.29 | NA | 6 | 1439435 |  |  |  |  |  |  |  |  |  |  |  |  |  |
| Log | (*N* = 8) | 232 | 0.45 | 0.00016 | 5 | 1319568 | 0.45 | 0.53 | Exp |  |  |  |  |  |  |  |  |  |  |
