# Supplementary material for: From virtually extinct to superabundant in 35 years: establishment, population growth and shifts in management focus of the Swedish wild boar (Sus scrofa) population
Source: BMC Zool. 2024 Jul 1;9:14. doi: 10.1186/s40850-024-00202-2 (PMC11218266; doi:10.1186/s40850-024-00202-2)
Supplement: Supplementary file 2 — Supplementary Material 2. Additional file 2. Swedish Code of Statue referenced in the text (Word file). [file 40850_2024_202_MOESM2_ESM.docx]

Additional file 2

**Swedish Code of Statues (SFS) referenced in the text**

|  |  |  |  |
| --- | --- | --- | --- |
| 1938:274 | 1947:402 | 1959:243 | 1978:776 |
| 1938:279 | 1947:403 | 1960:264 | 1981:175 |
| 1938:480 | 1948:397 | 1961:303 | 1981:177 |
| 1938:505 | 1949:359 | 1962:250 | 1985:823 |
| 1939:489 | 1950:305 | 1963:148 | 1987:259 |
| 1940:1 | 1951:412 | 1964:120 | 1987:905 |
| 1940:491 | 1952:415 | 1965:261 | 1988:1175 |
| 1940:740 | 1953:469 | 1966:282 | 1991:1770 |
| 1941:442 | 1953:606 | 1967:427 | 1994:1454 |
| 1941:443 | 1954:104 | 1967:773 | 1996:727 |
| 1942:559 | 1954:348 | 1968:355 | 1998:1000 |
| 1943:517 | 1955:94 | 1969:353 | 1999:657 |
| 1943:519 | 1955:377 | 1970:274 | 2000:592 |
| 1943:520 | 1955:595 | 1971:446 | 2002:551 |
| 1944:132 | 1956:56 | 1972:246 | 2008:1412 |
| 1944:350 | 1956:324 | 1973:406 | 2019:174 |
| 1945:399 | 1957:46 | 1974:554 | 2021:334 |
| 1946:391 | 1957:267 | 1975:543 | 2021:576 |
| 1946:393 | 1957:617 | 1976:432 | 2021:807 |
| 1947:95 | 1958:310 | 1977:327 |  |
