# Supplementary material for: From virtually extinct to superabundant in 35 years: establishment, population growth and shifts in management focus of the Swedish wild boar (Sus scrofa) population
Source: BMC Zool. 2024 Jul 1;9:14. doi: 10.1186/s40850-024-00202-2 (PMC11218266; doi:10.1186/s40850-024-00202-2)
Supplement: Supplementary file 3 — Supplementary Material 3. Additional file 3. Published scientific papers found in literature search (Word file). [file 40850_2024_202_MOESM3_ESM.docx]

Additional file 3

**Published research papers regarding the free-ranging wild boar population in Sweden, divided into seven categories**

The number of papers in each category in brackets

Health, including diseases (17)

Boqvist S, Bergström K, Magnusson U. 2012. Prevalence of antibody to six *Leptospira* servovars in Swedish wild boar. Journal of Wildlife Diseases 48(2): 492-496. <https://doi.org/10.7589/0090-3558-48.2.492>

Ernholm L, Sternberg-Lewering S, Ågren E, Ståhl K, Hultén C. 2022. First detection of *Salmonella* *enterica* serovar *choleraesuis* in free ranging European wild boar in Sweden. Pathogens 11(7), 723. <https://doi.org/10.3390/pathogens11070723>

Fabri N.D, Sprong H, Hofmeester T.R, Heesterbeek H, Donnars B.F, Widemo F, Ecke F, Cromsigt J.P.G.M. 2021. Wild ungulate species differ in their contribution to the transmission of *Ixodes* *ricinus*-borne pathogens. Parasites & Vectors 14: 360. <https://doi.org/10.1186/s13071-021-04860-w>

Jacobson M, Löfstedt M.G, Holmgren N, Lundeheim N, Fellström C. 2005 The prevalence of *Brachyspira* spp. And *Lawsonia* *intracellularis* in Swedish piglet producing heards and wild boar populations. Journal of Veterinary Medicine 52.9: 386-391. DOI: 10.1111/j.1439-0450.2005.00865.x

Jaenson T.G.T, Petersson E.H, Kindberg J, Pettersson J.H-O, Hjertqvist M, Medlock J.M, Bengtsson H. 2018. The importance of wildlife in the ecology and epidemiology of the TBE virus in Sweden: incidence of human TBE correlates with abundance of deer and hares. Parasites & Vectors 11: 477. <https://doi.org/10.1186/s13071-018-3057-4>

Malmsten A, Dalin A-M, Pettersson A. 2015. Caries, Peridontal disease, supernumerary teeth and other dental disorders in Swedish wild boar (*Sus* *scrofa*). Journal of Comparative Pathology 153:1. 50-57. <https://doi.org/10.1016/j.jcpa.2015.04.003>

Malmsten A, Magnusson U, Ruiz-Fons F, González-Barrio D, Dalin A-M. 2018. A sereologic survey of pathogens in wild boar (*Sus* *scrofa*) in Sweden. Journal of Wildlife Diseases 54(2): 229-237. DOI: 10.7589/2017-05-120

Malmsten A, Dalin A-M, Pettersson J, Persson S. 2021. Concentrations of cadmium, lead, arsenic, and some essential metals in wild boar from Sweden. European Journal of Wildlife Research 67: 18. <https://doi.org/10.1007/s10344-021-01460-y>

Pozio E, Christensson D, Stéen M, Marucci G, La Rosa G, Bröjer C, Mörner T, Uhlhorn H, Ågren E, Hall M. 2004. *Trichinella* *pseudospiralis* foci in Sweden. Veterinary Parasitology 125: 335-342. DOI: 10.1016/j.vetpar.2004.07.020

Roth A, Lin J, Magnius L, Karlsson M, Belák S, Widén F, Norder H. 2016. Markers for ongoing or previous *Hepatitis* E Virus infection are as common in wild ungulates as in humans in Sweden. Viruses 8:259. DOI: 10.3390/v8090259

Sannö A, Aspán A, Hestvik G, Jacobson M. 2014. Presence of *Salmonella* spp., *Yersinia* *enterocolitica*, *Yersinia* *pseudotuberculosis* and *Escherichia* *coli* O157:H7 in wild boars. Epidemiology & Infection 142: 2542-2547. DOI: 10.1017/S0950268814000119

Sannö A, Rosendal T, Aspán A, Backhans A, Jacobson M. 2018. Distribution of enteropathogenic *Yersinia* spp. and *Salmonella* spp. in the Swedish wild boar population, and assessment of risk factors that may affect their prevalence. Acta Veterinaria Scandinavia 60: 40. <https://doi.org/10.1186/s13028-018-0395-3>

Sannö A, Jacobson M, Sterner S, Thisted-Lambertz S, Aspan A. 2018. The development of a screening protocol for *Salmonella* spp. and enteropathogenic *Yersinia* spp. in samples from wild boar (*Sus* *scrofa*) also generating MLVA-data for *Y*. *enterocolitica* and *Y*. *pseudotuberculosis*. Journal of Microbiological Methods 150: 32-38. DOI: 10.1016/j.mimet.2018.05.014

Stenberg H, Leveringhaus E, Malmsten A, Dalin A-M, Postel A, Malmberg M. 2021. Atypical *Porcine* pestvirus – A widespread virus in the Swedish wild boar population. Transboundary and Emerging Diseases: 1 – 12. DOI: 10.1111/tbed.14251

Wallander C, Frössling J, Vågsholm I, Uggla A, Lundén A. 2015. *Toxoplasma gondii* seroprevalence in wild boars (*Sus scrofa*) in Sweden and evaluation of ELISA test performance. Epidemiology & Infection 143: 1913-1921. DOI: 10.1017/S0950268814002891

Wang H, Castillo-Contreras R, Saguti F, Lopez-Olvera J.R, Karlsson M, Mentaberre G, Lindh M, Serra-Cobo J, Norder H. 2019. Genetically similar hepatitis E virus strains infect both humans and wild boars in the Barcelona area, Spain, and Sweden. Transboundary and Emerging Diseases 66.2: 978-985. DOI: 10.1111/tbed.13115

Widen F, Sundqvist L, Matyi-Toth A, Metreveli G, Belak S, Hallgren G, Norder H. 2011. Molecular epidemiology of hepatitis E virus in humans, pigs and wild boars in Sweden. Epidemiology and Infection 139.3: 361-371. DOI: 10.1017/S0950268810001342

Reproduction (5)

Dalin A-M, Malmsten A, Jansson G. 2014. Reproductive seasonality among Swedish wild boars (*Sus* *scrofa*). Reproduction in Domestic Animals, Volume 49. 111 River St. Hoboken 07030-5774, NJ USA. Wiley-Blackwell.

Malmsten A, Dalin A-M, 2017. Puberty in female wild boar (*Sus* *scrofa*) in Sweden. Acta Veterinaria Scandinavica 58:1. DOI: 10.1186/s13028-016-0236-1

Malmsten A, Jansson G, Dalin A-M. 2017. Post-mortem examination of the reproductive organs of female wild boars (*Sus* *scrofa*) in Sweden. Reproduction in Domestic Animals 52:4. 570-578. <https://doi.org/10.1111/rda.12947>

Malmsten A, Jansson G, Lundeheim N, Dalin A-M. 2017. The reproductive pattern and potential of free ranging wild boars (*Sus* *scrofa*) in Sweden. Acta Veterinaria Scandinavia 59: 52: <https://doi.org/10.1186/s13028-017-0321-0>

Bergqvist G, Paulsen S, Elmhagen B. 2018. Effects of female body mass and climate on reproduction in northern wild boar. Communication. Wildlife Biology: 1-6. DOI: 10.2981/wlb.00421

Movements, including home ranges and natal dispersal (6)

Lemel J, Truvé J, Söderberg B. 2003. Variation in ranging and activity behaviour of European wild boar *Sus* *scrofa* in Sweden. Wildlife Biology 9(4): 29-36. <https://doi.org/10.2981/wlb.2003.061>

Muthoka C.M, Andrén H, Nyaga J, Augustsson E, Kjellander P. 2022. Effect of supplemental feeding on habitat and crop selection by wild boar in Sweden. Ethology, Ecology & Evolution. DOI: 10.1080/03949370.2021.2024265

Thurfjell H, Ball J.P, Åhlén P-A, Kornacher P, Dettki H, Sjöberg K. 2009. Habitat use and spatial patterns of wild boar *Sus* *scrofa* (L.): agricultural fields and edges, European Journal of Wildlife Research 55: 517-523. DOI: 10.1007/s10344-009-0268-1

Thurfjell H, Spong G, Ericsson G. 2014. Effects of weather, season and daylight on female wild boar movement. Acta Theriologica 59: 467-472. <https://doi.org/10.1007/s13364-014-0185-x>

Truvé J, Lemel J. 2003. Timing and distance of natal dispersal for wild boar *Sus* *scrofa* in Sweden. Wildlife Biology 9(4): 51-57. <https://doi.org/10.2981/wlb.2003.056>

Truvé J, Lemel J, Söderberg B. 2004. Dispersal in relation to population density in wild boar (*Sus* *scrofa*). Galemys 16: 75-82. ISSN 1137-8700.

Feeding, including rooting and damages (5)

Brunet J, Hedwall P-O, Holmström E, Wahlgren E. 2016. Disturbance of the herbaceous layer after invasion of an eutrophic temperate forest by wild boar. Nordic Journal of Botany 34:1. 120-128. <https://doi.org/10.1111/njb.01010>

Carpio A.J, Hillström L, Tortosa F.S. 2016. Effects of wild boar predation on nests of wading birds in various Swedish habitats. European Journal of Wildlife Research. DOI: 10.1007/s10344-016-1016-y

Carpio A.J, Garcia M, Hillström L, Lönn M, Carvalho J, Acevedo P, Bueno C.G. 2022. Wild boar effects on fungal abundance and guilds from sporocarp sampling in a boreal forest ecosystem. Animals 12: 2521. <https://doi.org/10.3390/ani12192521>

Gren I-M, Andersson H, Mensah J, Pettersson T. 2020. Cost of wild boar to farmers in Sweden. European Review of Agriculture Economics 47: 1. 226 – 246. <https://doi.org/10.1093/erae/jbz016>

Welander J. 2001. Spatial and temporal dynamics of wild boar (*Sus* *scrofa*) rooting in a mosaic landscape. Journal of Zoology 252(2): 263-271. DOI: <https://doi.org/10.1111/j.1469-7998.2000.tb00621.x>

Hunting (8)

Bergqvist G. 2022. Harvest bag composition differs among hunting methods for wild boar in Sweden. Short communication. European Journal of Wildlife Research 68:27. <https://doi.org/10.1007/s10344-022-01576-9>

Engelman M, Lagerkvist C-J, Gren I-M. 2018. Hunters´ trade-off in valuation of different game animals in Sweden. Forest Policy and Economics 92: 73-81. <https://doi.org/10.1016/j.forpol.2018.04.004>

von Essen E. 2020. How wild boar hunting is becoming a battleground. Leisure Sciences 42: 5-6. DOI: 10.1080/01490400.2018.1550456

Fahlman Å, Lindsjö J, Arvén Norling T.A, Kjellander P, Ågren E.O, Bergvall U.A. 2020. Wild boar behaviour during live-trap capture in a corral-style trap: implications for animal welfare. Acta Veterinaria Scandinavia 62: 59. <https://doi.org/10.1186/s13028-020-00557-9>

Fahlman Å, Lindsjö J, Bergvall U.A, Ågren E.O, Norling T.A, Stridsberg M, Kjellander P, Höglund O. 2021. Measurement of *Catestatin* and *Vasostatin* in wild boar *Sus scrofa* captured in a corral trap. *BMC Research Notes* 14, 337. <https://doi.org/10.1186/s13104-021-05742-1>

Gentsch R.P, Kjellander P, Röken B.O. 2018. Cortisol response of wild ungulates to trauma situations: hunting is not necessarily the worst stressor. European Journal of Wildlife Research 64: 11.  <https://doi.org/10.1007/s10344-018-1171-4>

Mensah J.T, Elofsson K. 2017. An empirical analysis of hunting lease pricing and value of game in Sweden. Land Economics 93:2. 292-308. DOI: 10.3368/le.93.2.292

Thurfjell H, Spong G, Ericsson G**.** 2013. Effects of hunting on wild boar *Sus* *scrofa* behaviour. Wildlife Biology 19(1): 87-93. <https://doi.org/10.2981/12-027>

Traffic (2)

Jägerbrand A, Gren I-M. 2018. Consequences of increases in wild boar – vehicle accidents 2003 – 2016 in Sweden on personal injuries and costs. Safety 4. DOI: 10.3390/safety4040053

Thurfjell H, Spong G, Olsson M, Ericsson G. 2015. Avoidance of high traffic levels results in lower risk of wild boar-vehicle accidents. Landscape and Urban Planning 133: 98-104. <https://doi.org/10.1016/j.landurbplan.2014.09.015>

Population (1)

Gren I-M, Häggmark-Svensson T, Andersson H, Jansson G, Jägerbrand A. 2015. Using traffic data to estimate wildlife populations. Journal of Bioeconomics 18(1): 17-31. DOI: 10.1007/s10818-015-9209-0
